# Bolivia’s Path to Lower Emissions: Sugar Cane Ethanol and GREET Model Insights into Ethanol-Gasoline Blends

**DOI:** 10.1021/acsomega.5c00346

**Published:** 2025-06-12

**Authors:** Victor S. V. Mercado, Romilda Fernandez-Felisbino, Jean F. Leal Silva, Laura Plazas Tovar

**Affiliations:** † Department of Chemical Engineering, 505146 Federal University of São Paulo, Rua Prof. Artur Riedel, 275 - Eldorado, Diadema, SP 09972-270, Brazil; ‡ School of Chemical Engineering, 28132 University of Campinas, Avenida Albert Einstein 500−Cidade Universitária, Campinas, SP 13083-852, Brazil

## Abstract

Bolivia’s transition toward sustainable energy is advancing through the integration of sugar cane ethanol as a viable alternative to fossil fuels. This study presents the first gate-to-gate (GtG) life cycle assessment (LCA) of Bolivian ethanol production, developed using Aspen Plus v14 with a base unit of 1 MJ of energy. The assessment considers a sugar cane milling capacity of 23,000 tons per day and an annual production potential of 108 million liters of anhydrous ethanol. Distillation emerged as the most significant contributor to the GtG global warming potential (GWP) at 31.0 gCO_2_eq/L; however, ethanol’s life-cycle emissions were significantly lower (1.61 gCO_2_eq/MJ) than gasoline (70.74 gCO_2_eq/MJ). Complementarily, a tank-to-wheel (TtW) LCA was conducted using the GREET model and a functional unit of 1 km traveled, to evaluate the impact of ethanol-gasoline blends on Bolivia’s national vehicle fleet from 2022 to 2028. Results show that an E25 blend could reduce GHG emissions by up to 11.2% in passenger cars, 13.2% in SUVs, and 15.2% in pick-up trucks by 2028. Significant reductions in carbon monoxide (25.1%) and volatile organic compounds (21.0%) also enhance air quality. Economic analysis demonstrated a net present value of 202.94 million USD and an internal rate of return of 7.93%, reinforcing ethanol’s profitability and strategic value in Bolivia’s low-carbon transition.

## Graphical abstract

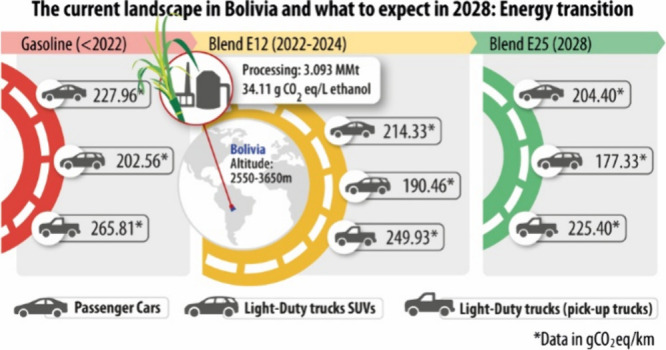

## 1 Introduction

The global drive for decarbonization has intensified as nations align with the Paris Climate Agreement to combat climate change. A major challenge is the transformation of energy systems, particularly in developing countries that depend on fossil fuels.^1–3^ Although the environmental and economic aspects of ethanol production have been investigated over the last 15 years in countries such as Brazil and the United States,^4,5^ these studies largely reflect the context in which mature ethanol markets, well-developed infrastructure, and stable policy frameworks are already in place. Bolivia, in contrast, presents a distinctive combination of high-altitude terrain (2550–3650 m), landlocked geography, variable climate conditions, and an emerging policy environment that heavily subsidizes raw materials.^2^

The agricultural landscape of Bolivia is ideal for sugar cane cultivation, with a forecast crop area of 307,000 ha by 2025, yielding 17 million tons.^6^ This supports Bolivia’s shift from fossil fuel reliance to ethanol, a cleaner fuel, for transportation sector (the second-largest source of greenhouse gas (GHG) emissions),^7,8^ which accounts for 19% of Bolivia’s total emissions, producing about 13.9 MtCO_2_eq annually.^9,10^ Bolivia’s reliance on natural gas and the early stage of its ethanol industry offers distinct opportunities for global decarbonization efforts if addressed through targeted research and policies.^11^ These circumstances offer a novel lens through which to analyze the life-cycle emissions and profitability of sugar cane-derived ethanol.

Our previous study (Mercado et al.^11^) addressed the agricultural stage of sugar cane ethanol production. We collected farm-level data on fertilizer use, crop yields, and field operations in Santa Cruz, Bolivia, estimating a cradle-to-farm-gate carbon intensity of 21.48 kgCO_2_eq per metric ton of sugar cane in the 2022/2023 season. Nitrous oxide emissions from fertilizer application and energy inputs for cultivation and harvesting were the largest contributors to the cradle phase. Results from Monte Carlo simulations and projected yield improvements suggest that greater adoption of precision agriculture practices may reduce these emissions by as much as 15% over the coming years.

Sugar cane ethanol offers lower GHG emissions than gasoline. In South America, ethanol is key in energy policies for lowering fuel global warming potential (GWP).^12–15^ Brazil’s RenovaBio program aims for an 11% GHG reduction in fuels by 2030.^16,17^ Ecuador requires a 5% ethanol blend, targeting a GWP of 130–180 gCO_2_eq/km.^18^ Argentina’s Biofuel Law 27640 mandates a 12% blend, cutting GWP by up to 70% against gasoline’s 87.4 gCO_2_eq/MJ.^16^ Colombia’s ethanol blend was adjusted to 4% by 2023, yet an E10 blend achieves a lower GWP (88.1 gCO_2_eq/MJ) than gasoline’s 94 gCO_2_eq/MJ.^16,19^

Although countries such as Brazil and Argentina have successfully integrated ethanol into their transportation fuels, Bolivia’s journey toward leveraging its sugar cane ethanol potential is just beginning. Bolivia’s unique high-altitude geography (2550–3650 m above sea level) and diverse climate zones pose specific challenges and opportunities for ethanol production, which require tailored research.^20,21^ Since 2018, Bolivia has implemented biofuel initiatives, starting with a 10% ethanol-gasoline blend (E10) and aiming for a 25% blend (E25) by 2028 (Law 1098 of 2018).^22^ In March 2024, the Bolivian government reaffirmed the 12% ethanol blend (E12) (Supreme Decree 3672). These blends diversified Bolivia’s energy portfolio, improved energy security, and strengthened the agro-industrial economy, particularly benefiting sugar cane mills that are crucial for rural development.^11^ Moreover, the impact of sugar cane ethanol on GHG emissions is contingent upon ethanol source, feedstock carbon intensity, production efficiency, and vehicle use.^19,23^

Despite the initial steps toward integrating ethanol into its energy mix, Bolivia faces unmet needs in fully assessing the environmental life cycle and economic viability of sugar cane ethanol production at a national scale. Addressing these knowledge and implementation gaps is crucial for Bolivia’s shift to low-carbon fuels and for meeting broader environmental and economic goals, such as climate action (SDG 13) and economic growth (SDG 8).

In this context, the present study conducted a gate-to-wheel life cycle assessment (LCA) and techno-economic evaluation of sugar cane ethanol production in Bolivia, integrating site-specific industrial data from the country’s largest ethanol facility with GREET-based modeling to capture altitude-related emission effects. By systematically combining production data (e.g., bagasse cogeneration, distillation, fermentation, and dehydration) with a tailored assessment of Bolivia’s vehicle fleet and policy-driven blending targets, we provide an original perspective on the interplay between resource constraints, operational parameters, and government incentives. This integrated framework offers insights not only for Bolivia but also for similar countries looking to balance rural development, energy security, and greenhouse gas (GHG) mitigation, bridging a critical knowledge gap in the broader ethanol research landscape. The findings reveal the key drivers for optimizing sugar cane-based ethanol, highlighting its potential to reduce reliance on imported fossil fuels, while contributing to global decarbonization objectives.

## 2 Materials and Methods

This study provides the first gate-to-gate (GtG) life cycle assessment (LCA) of ethanol production in Bolivia. Additionally, a tank-to-wheel (TtW) LCA was carried out to complement the analysis to assess the GWP of ethanol in Bolivia under current policies. The life-cycle system boundaries include agricultural production, industrial production, and fuel consumption stages, as shown in Figure 1.

**Figure 1:**
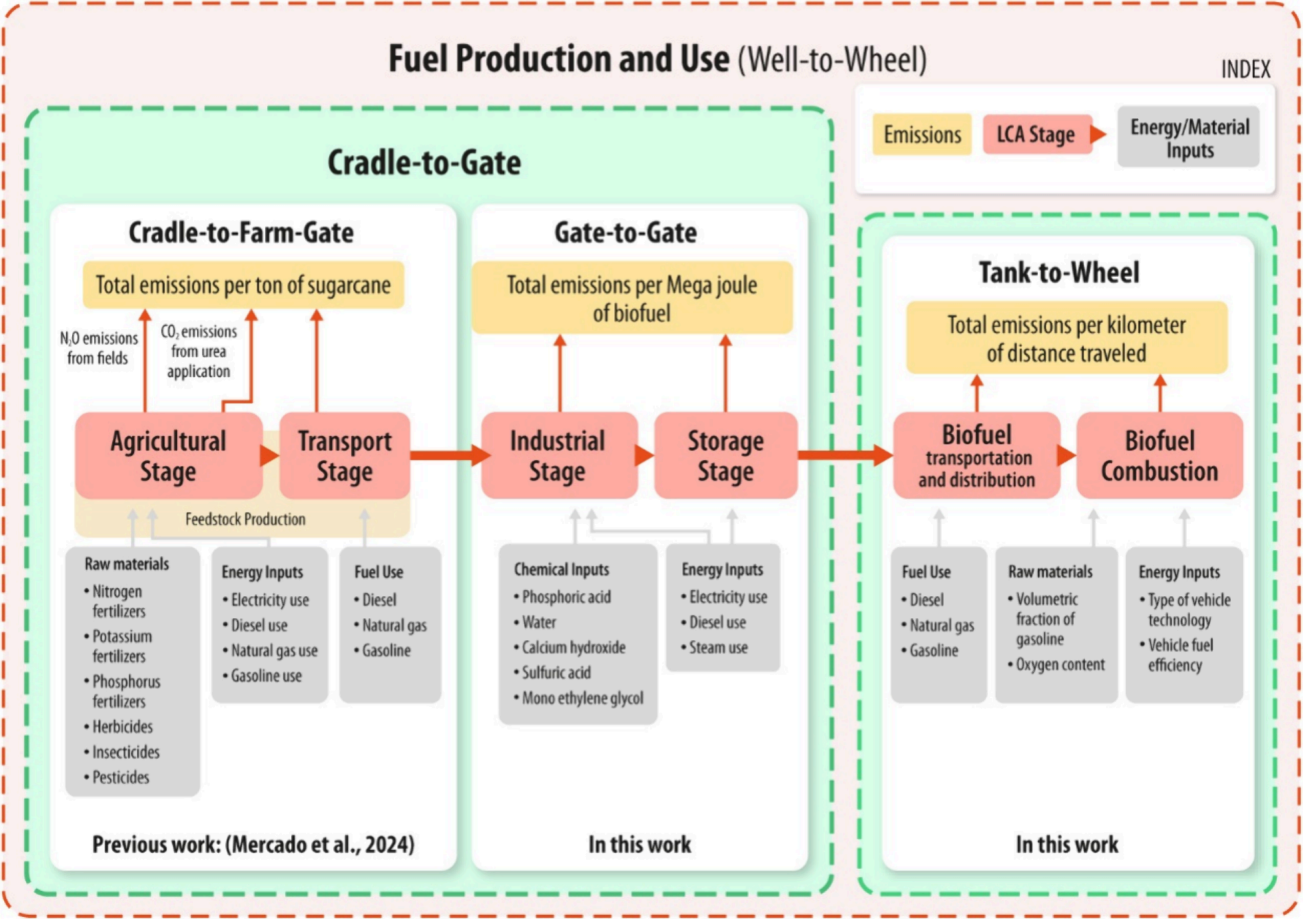
Life-cycle system boundaries for Bolivia’s sugar cane ethanol.

### 2.1 A Gate-to-Gate Life Cycle Assessment for Ethanol Production

#### 2.1.1 System Boundaries, Scope, and Functional Unit

The first analysis investigated the sugar cane-to-ethanol production processes at the Guabirá distillery in Santa Cruz, Bolivia (17°18′55.6″S 63°15′44.6″W), focusing on sugar cane milling, fermentation, distillation, and dehydration, while excluding upstream (e.g., agricultural inputs for sugar cane cultivation) and downstream (e.g., ethanol distribution, consumption, and disposal) activities (Figure 2a) on a gate-to-gate (GtG) LCA approach. The Guabirá plant, which is crucial for ethanol production, energy independence, and decarbonization efforts in Bolivia, has achieved new records in 2023, processing 23,000 tons of sugar cane and producing approximately one million liters of ethanol daily during the 148-day annual sugar cane harvesting season. The plant also generates renewable energy from bagasse combustion, supplying 64,000 MWh to the National Integrated System (SIN) in 2023, reducing natural gas use in electricity production. The LCA was evaluated using a consistent functional unit of ethanol energy content (1 MJ).

**Figure 2:**
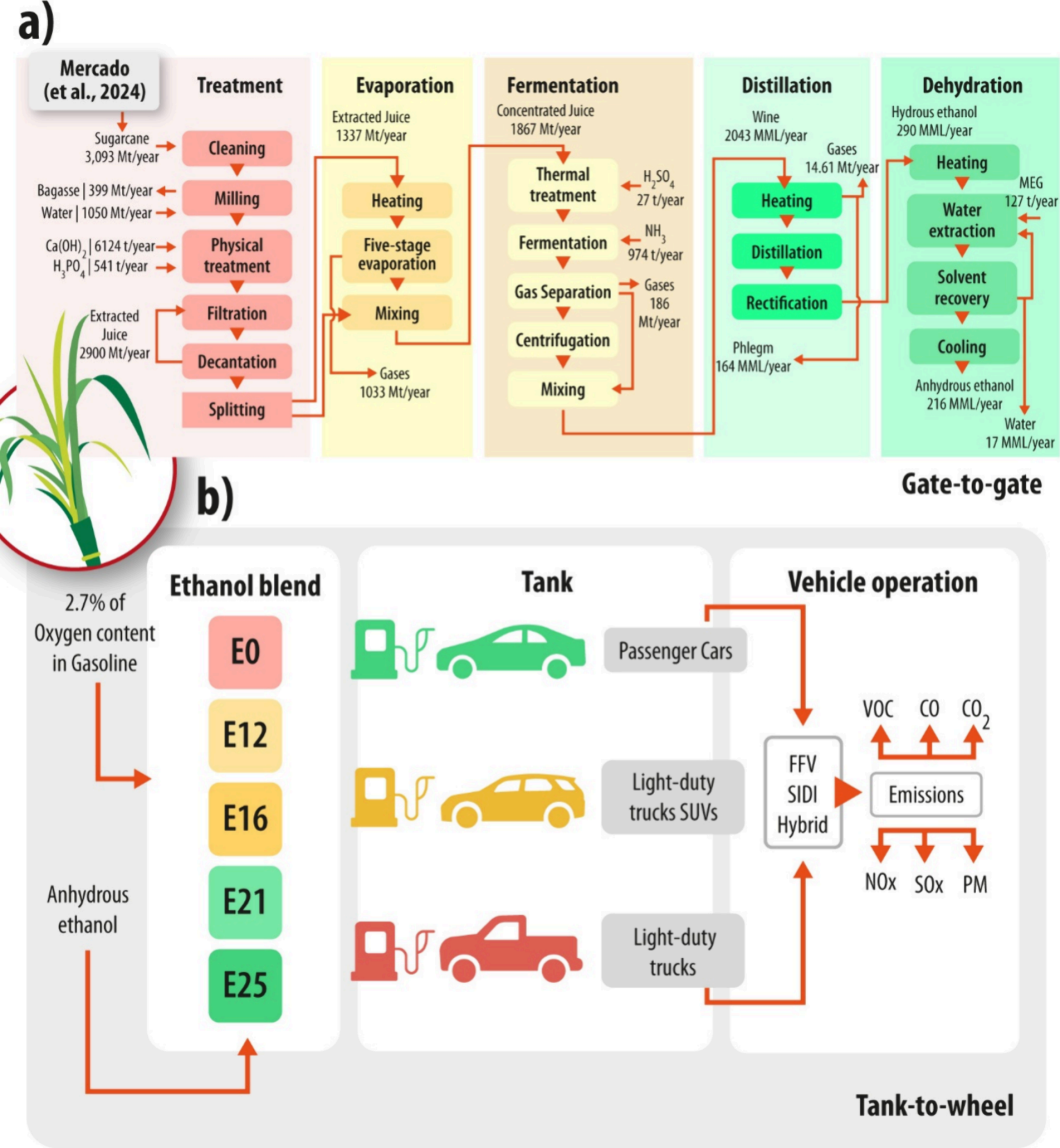
Life-cycle system boundaries used in this study. Foreground systems incorporate both ethanol production and its use in vehicle passenger cars, light-duty trucks, SUVs (SUVs and CUVs), and light-duty trucks (specifically, pick-up trucks). The vehicle technologies considered include flexible fuel vehicles (FFV), spark ignition and direct injection (SIDI), and hybrid vehicles (HV): (a) Gate-to-Gate system boundary and (b) Tank-to-Wheel system boundary.

#### 2.1.2 Inventory Data Collection

Life cycle inventory (LCI) data were collected for the key inputs and outputs.

- Process inputs: Data from the Guabirá plant, Bolivia’s largest ethanol producer, and sugar cane ethanol production results from Aspen Plus v14 simulation were used.
- Energy inputs: This process relies solely on bagasse combustion, enabling the facility to supply 21 MW to the SIN.^7^ Bagasse from milling was fed into a combustion boiler to produce high-pressure steam for electricity generation and mill energy supply. Consequently, the energy of the process depends on the biomass.
- Raw material inputs: In 2023, Guabirá processed 23,000 tons of sugar cane daily, producing approximately 108 million liters. This production determines the quantities of the raw materials, utilities, and byproducts. Nonetheless, the Bolivian government is responsible for the production of sugar cane and provides a subsidized provision (at no cost) to any company that can produce a manufactured good from sugar cane, as long as the country’s food security is guaranteed (Law 307 and Supreme Decrees 1554 and 3456), encouraging the production of sugar and ethanol. The chemical inputs of LCI in the GtG assessment are listed in Table S8 (Supporting Information, Section S3).
- Emissions: Direct GHG emissions were calculated based on the assumption that all carbon was converted into biogenic CO_2_ and were determined from the fermentation reaction, where CO_2_ is a byproduct. Utility consumption emissions were calculated using a bagasse combustion simulation. The emissions of CH_4_ and NO_*x*_ were calculated using the emission factors from US Environmental Protection Agency (EPA) Rule-E9-5711 for biomass combustion, as biomass was not included in the Aspen Plus v14 databank.
- Waste streams: Bagasse is treated as fuel for steam generation and as a coproduct. The filter cake and vinasse (liquid residue from distillation) were also quantified.

#### 2.1.3 Process Simulation and Data Sources

Gate-to-gate inventory includes process boundaries, chemical reactions and yields, mass balances, material inputs and outputs, energy consumption for various operations, energy sources, and potential energy losses. Aspen Plus v14 was used to simulate the production process. Sugar cane feedstock consisted of both conventional and hypothetical components. Biomass components, such as cellulose, hemicellulose, and lignin, were defined based on previous literature and modeled as solids, as detailed in Table S1 (Supporting Information, Section S1). Yeast properties were derived from Da Luz et al.,^14^ Oliveira and Cruz.^12^ and Santoyo-Castelazo et al.^13^ (detailed in Table S2). The thermodynamic models used were NRTL-RK, which deals with the phase equilibrium of nonideal solutions, and UNIQUAC, because empirical data on the binary interaction parameters of sucrose and water in the liquid phase are available. The ethanol production process in the Guabirá plant was divided into five stages: (S1) treatment, (S2) evaporation, (S3) fermentation, (S4) distillation, and (S5) dehydration. A cogeneration system was also considered (see Supporting Information, Section S2).

##### (S1) Treatment

Sugar cane treatment entails cleaning, extracting, and physically processing the juice. Initially, sugar cane was cleaned to remove 70% of debris, soil, and minerals using a *component separator* model, then mixed with hot water (28% of the product stream) and processed through a second *component separator model* to remove 20% solids.^12,24^ The juice was then extracted (simulated as a *component separator* model) to remove 98% of bagasse, which was then sent to a boiler for electricity generation. After extraction, the juice was heated to 70 °C and treated with phosphoric acid and calcium hydroxide for flocculation, which was simulated using the *RStoic* model. The stream was further heated to 105 °C and sent to a separator (*flash* model) to release volatile gases, and then to a decanter (*component separator* model) to remove 100% solids, 17% sucrose, and glucose. To recover the sugars carried by the solid fraction, a second decanter was used and the product stream was mixed with the main juice stream for the next stage.

##### (S2) Evaporation

Part of the juice was concentrated and heated using a five-effect evaporator system, with each effect operating at different pressures and temperatures, from a temperature of 115.6 °C and a pressure of 1.7 bar in the first stage to 60.4 °C and 0.2 bar in the fifth stage, increasing the juice’s Brix to 65°. A combination of *flash*, *valve,* and *HeatX* models was used to simulate the evaporators (see Figure S1). The concentrated juice was then mixed with the remaining fraction to obtain a juice of 20° Brix for fermentation.

##### (S3) Fermentation

The concentrated juice was sterilized at 130 °C and then cooled to 32 °C for optimal fermentation. An *RStoic* model at 32 °C and atmospheric pressure was used to simulate the fermentation reactions that convert sucrose to glucose and fructose, followed by the conversion of glucose to ethanol. Simulated byproducts of yeast metabolism included glycerol, succinic acid, acetic acid, and isoamyl alcohol. Carbon dioxide produced during fermentation was removed from the reactor outlet stream (*Flash2* model), and passed through a ten-stage absorber column (*RadFrac* model) to recover ethanol. The liquid stream from the fermenter was centrifuged in a *CFuge* model to separate the yeast, which was then directed to a second *CFuge* that guaranteed ethanol recovery and recirculated the solid fraction (yeast) into the fermenter. The first *CFuge* liquid outlet was mixed with the liquid outlet from the second *CFuge* model and was sent to the distillation stage.

##### (S4) Distillation

This stage was modeled as a combination of *RadFrac* columns. Table S5 provides additional details of the four distillation columns. The fermentation broth was heated to 93 °C in a *HeatX* model and entered the first section of the first distillation column A1, with the bottom-stream product feeding column D and top stream product feeding column A. In columns A and D, the reflux ratio and reboiler duty were adjusted to obtain the desired ethanol concentrations. In column D, the gas outlet, which contained 90%wt of CO_2_, was cooled to 35 °C to avoid ethanol loss. The top outlet of A and the bottom outlet of D were mixed and fed into column B–B1, with the reflux ratio and reboiler duty adjusted to achieve 90%wt of ethanol (hydrated ethanol) at the top outlet and <0.002%wt at the bottom outlet.

##### (S5) Dehydration

Hydrated ethanol enters the *HeatX* model to exchange heat with the stripping component, monoethylene glycol (MEG). The hydrated ethanol stream, at 130 °C and 1.02 bar, feeds an extractive distillation column where it contacts MEG, which alters the azeotropic condition and directs water to the column’s bottom, while the dehydrated ethanol stream (99.8%wt) exits from the top. The MEG and water mixture proceeded to a recovery column, simulated as a *RadFrac* model at 158 °C and 0.3 bar, producing a water stream at the top and recovering MEG with over 98% purity at the bottom. The bottom stream was pumped, mixed with makeup, and returned to the extractive distillation column. Additional details for both columns are provided in Table S6.

#### 2.1.4 Impact Assessment

This study assessed GWP by treating biogenic CO_2_ emissions as carbon-neutral, including CO_2_ emissions from bagasse combustion and auxiliary fossil fuel use. The simulation environment, calculation of emissions based on energy sources, and utility consumption using the US-EPA-Rule-E9-5711 emission factors make the GWP the only impact assessment category investigated.

### 2.2 Economic Feasibility

Two scenarios were analyzed for economic evaluation: SB1, without production expansion, and SB2, which assumes production expansion until 2032 based on 2021–2022 Bolivian contract trends. Tables S9 and S10 (Supporting Information, Section S4) present the corresponding annual production and ethanol price, respectively. The evaluation incorporated a 25-year plant lifespan, 45% income tax rate, and 12.5% annual depreciation rate. As previously stated, the government ensures the supply of sugar cane for both crystallized sugar and ethanol production. To account for this circumstance, we consider the cost of sugar cane only during the 2033–2045 period, while this cost is not factored in for the 2021–2032 period.

#### 2.2.1 Economic Assumptions and Analysis of Profitability

Mass balance simulation results were used to determine the raw material costs and primary inputs (Tables S8 and S10). The utility costs encompass water for cooling and steam generation given the plant’s energy self-sufficiency. Currently, there is no market for electricity generated from bagasse combustion; therefore, potential electricity revenue is excluded.

Equipment investment (EI) was estimated using Aspen Plus v14 and Aspen Economic Analyzer, considering operation and usage. The fixed capital investment (FCI) and total capital investment (TCI) were calculated as shown in Table S10. The cash flow analysis method was used to analyze profitability based on net present value (NPV), payback time, internal rate of return (IRR), and minimum ethanol selling price (MESP).

#### 2.2.2 Sensitivity Analysis

The sensitivity analysis aimed to investigate the influence of various process parameters on the NPV and MESP calculated in the economic analysis. The ethanol price, equipment investment, and costs of raw materials, utilities, and labor were independently evaluated with ± 10, 20, and 30% variations, respectively.

### 2.3 Tank-to-Wheel Life Cycle Assessment

#### 2.3.1 Study Scope and System Boundary

A Tank-to-Wheel (TtW) LCA of fuel consumption in Bolivia’s road transportation sector was conducted, focusing on GWP, air pollutants, and fuel use during vehicle operation, excluding upstream processes (Figure 2b). It examines typical Bolivian fuel blends, such as E12, E16, E21, and E25, following national regulations and includes 100% fossil-based gasoline for comparison. This study evaluated various vehicles by considering the local vehicle fleet characteristics, fuel quality standards, driving conditions, and usage patterns specific to Bolivia. The base unit for the TtW LCA was 1 km of vehicle distance traveled.

#### 2.3.2 Tank-to-Wheel Inventory and Functional Unit

Bolivia’s vehicle fleet was analyzed with a focus on seven categories: automobiles, vans, jeeps, minibuses, pick-up trucks, sport utility vehicles (SUVs), and crossover utility vehicles (CUVs) (Supporting Information, data set S1). By employing the GREET parameters (version v1.3.0.14168, Argonne National Laboratory), these categories were classified as passenger cars (automobiles, vans, jeeps, and minibuses), light-duty trucks SUVs (SUVs and CUVs), and light-duty trucks (involving only pick-up trucks), collectively representing 55% of the fleet as internal combustion engine vehicles (ICEVs).^25^ The vehicle technologies considered include flexible fuel vehicles (FFV), spark-ignition, direct-injection (SIDI), and hybrid vehicles (HV).

The Bolivian ethanol blend policies affected 52.4% of the vehicle fleet (Supporting Information, data set S1). This study investigated the gasoline-ethanol blend and its fuel pathway using a functional unit of 1 km of distance traveled per vehicle and an energy base of 1 MJ. Bolivia’s decarbonization scenarios proposed ethanol blends of E12 in 2024, E16 in 2025, E21 in 2026, and E25 in 2027 and 2028 while maintaining a 12% blend in March 2024.

#### 2.3.3 Data Collection, Input Assumptions, and Modeling

In this study, facility-level information from the Guabirá distillery (including daily sugar cane throughput, bagasse combustion rates, and actual fermentation outputs) was integrated with nationally reported energy statistics and transportation-sector data (covering vehicle efficiency, annual mileage, and altitude adjustments). By combining these local production metrics with broader fleet usage parameters, the GREET model inputs accurately captured the reality of Bolivian ethanol supply and demand.

Where local data were unavailable, default values from the GREET model were adapted to the Bolivian conditions.

Vehicle fuel efficiency: Official Bolivian transportation databases provided fuel economy data (L/100 km) for each vehicle category, with the average gasoline consumption assumed to be 9.64 L/100 km for passenger cars, 8.35 L/100 km for light-duty trucks SUVs, and 10.62 L/100 km for light-duty trucks.^20^
Fuel characteristics: The oxygen content of the Bolivian gasoline was 2.7 wt %. The carbon intensity of gasoline was modeled using the default GREET assumptions.
The default emission factors for CO_2_, CH4, VOC, NO_*x*_, SO_*x*_, and particulate matter (PM) from the GREET database were used.

#### 2.3.4 GREET Model Configuration and Modifications

The GREET model of the Argonne National Laboratory^26^ was used for the LCA. The GREET model was selected because its extensive database, open-source nature, and adaptable framework enable the comprehensive incorporation of Bolivian-specific parameters, including altitude-related factors, into the life cycle assessment of sugar cane ethanol as follows:

- Vehicle operation conditions: Average speed, driving patterns, and load conditions in urban and rural Bolivian settings from transportation surveys, reflecting 60% of urban and 40% of rural driving cycles.
- Fuel distribution and consumption: The average fuel consumption rate and the 15,000 km annual travel distance for the three categories of vehicles in Bolivia were included in the TtW calculations.
- Altitude adjustment: Fuel consumption and emissions were adjusted for Bolivia’s altitude, particularly La Paz (3,650 m), with a 10–15% increase owing to reduced oxygen levels affecting the combustion efficiency.

#### 2.3.5 Impact Assessment

The TtW LCA evaluated GHG emissions (CO_2_, CH_4_, and N_2_O) in CO_2_ equivalents (CO_2_eq) and pollutants of local impact (VOC, CO, NO_*x*_, SO_*x*_, PM10, and PM2.5) for ethanol-gasoline blends. It assessed the Bolivian fuel economy, reflecting energy consumption (MJ/km) from both fossil and renewable sources. The factors considered included vehicle technology adoption rates, travel distances, fuel efficiency, and altitude adjustments.

## 3 Results and Discussion

### 3.1 Previous Report on Cradle-to-gate Emissions

Incorporating cradle-to-gate emissions is essential to accurately quantify the total life-cycle impact of Bolivian sugar cane ethanol. Although this work centers on the industrial processing phase (milling, fermentation, distillation) and eventual ethanol use (tank-to-wheel), the upstream farming contributions reported by Mercado et al.^11^ underscore the overall improvement opportunities across the entire value chain:

- Fertilizers and field-level N_2_O emissions represent a substantial share of cradle-stage emissions (41%). Improved soil management and fertilizer application techniques (e.g., split applications, nitrification inhibitors, or precision farming) can lower the total carbon footprint of ethanol, thereby enhancing the benefits of ethanol blends in reducing transport sector emissions.
- The prospective yield increase to nearly 59.3 t/ha by 2028 indicates that sustained agronomic gains, through superior crop varieties, efficient irrigation, and modern agricultural practices, can substantially reduce the carbon dioxide footprint up to 15% (18.2 kgCO_2_eq per ton of sugar cane). As sugar cane cultivation expands to meet the rising ethanol demand, maintaining high yields will be pivotal in maintaining land-use change impacts and the overall GHG intensity under control.
- The feedstock carbon intensity of 21.48 kgCO_2_eq per ton of sugar cane is directly fed into the broader life cycle calculation. Even modest improvements at this stage favorably impact the well-to-wheel emissions of ethanol, amplifying the total climate benefit when ethanol is blended with gasoline.

### 3.2 GWP of Bolivian Sugar Cane Ethanol Production

The case study of the Guabirá distillery in Bolivia yielded 61 m^3^/h of ethanol. Results of the environmental impact assessment for the GtG LCA show that the GWP is 1.61 gCO_2_eq/MJ, being distillation the highest contributor (31.0 gCO_2_eq/L) (Figure 3a), highlighting a significant reduction in comparison with gasoline.

**Figure 3:**
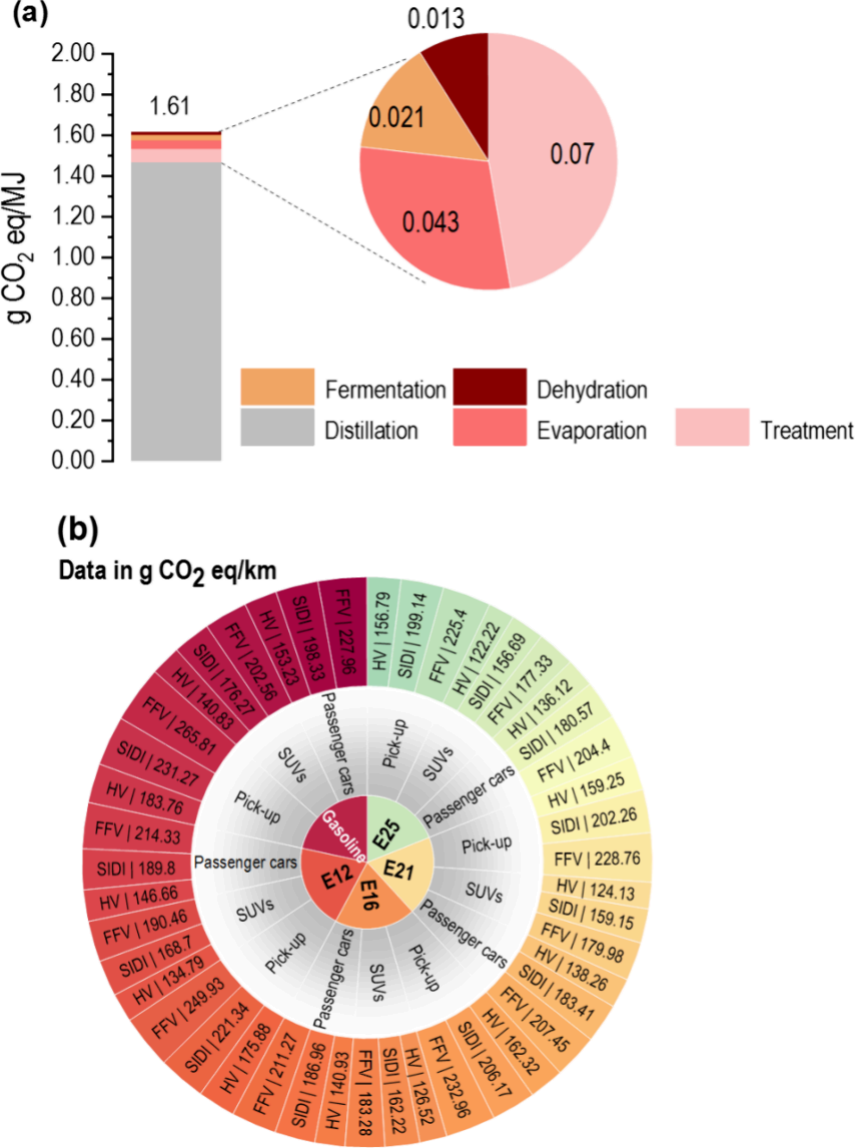
(a) Global warming potential (gCO_2_eq/MJ of processed sugar cane bagasse) associated with ethanol production. (b) Global warming potential for fuel in Bolivia’s transition from gasoline to 2022 and ethanol blends of E12 in 2024, E16 in 2025, E21 in 2026, and E25 in 2027 and 2028. Various vehicle categories were analyzed: passenger cars, light-duty trucks, SUVs (SUVs and CUVs), and light-duty trucks (specifically, pick-up trucks). The vehicle technologies considered include flexible fuel vehicles (FFV), spark ignition and direct injection (SIDI), and hybrid vehicles (HV).

The comparative data showed the potential of Bolivia for sustainable ethanol production. Canabarro et al.^16^ reported that Argentina’s ethanol production reduced GHG emissions by 70% compared to gasoline, with a GWP of 87.40 gCO_2_eq/MJ. Jozami et al.^27^ calculated a GtG GWP between 18 and 75 gCO_2_eq/MJ for second-generation ethanol production from Argentinian perennial grass. The differences in the GtG GWP are due to the fact that both studies considered the Argentine energy matrix (which is more than 50% dependent on fossil fuels), and the Bolivian case (1.61 gCO_2_eq/MJ) depends entirely on the burning of sugar cane bagasse favoring low GHG emissions and decarbonization goals.

Santoyo-Castelazo et al.^13^ reported that ethanol production from gasified sugar cane bagasse in Mexico had a GWP of 0.63 kgCO_2_eq/L in sugar cane treatment and 6.75 kgCO_2_eq/L in industrial production, considering that 94% of the energy supplied to the ethanol production process comes from fossil fuels (Mexican energy matrix). In Bolivia, our findings show 34.11 gCO_2_eq/L of ethanol, indicating an efficient production process from a GtG perspective. However, this analysis excludes upstream activities, such as feedstock cultivation and transportation, which significantly impact the total life-cycle emissions.^11^

Bolivia’s ethanol production exhibited a competitive GWP (1.61 gCO_2_eq/MJ) compared to global data. Oliveira and Cruz.^12^ estimated a GWP between 0.90 and 0.97 gCO_2_eq/MJ in a sugar cane mill in Brazil, whereas Liu et al.^15^ estimated a GWP in the same country of 1.1 gCO_2_eq/MJ using the GREET model. Both studies considered the Brazilian scenario, the dependence of the energy matrix on the burning of sugar cane bagasse, and the fact that biogenic carbon emissions (carbon naturally contained in biomass) do not affect the GWP of biofuel.

Arcentales-Bastidas et al.^18^ found a GWP of 0.32 kgCO_2_eq/L for ethanol in Ecuador considering that the energy matrix was composed of 92% hydroelectric energy and 8% of autonomous thermal generation. Rueda Ordoñez et al.^28^ reported a GWP between 0.4 and 1.3 kgCO_2_eq/L for a GtG LCA in Colombia, considering a scenario with an energy matrix dependent on coal combustion and another scenario in which all energy is provided by the combustion of sugar cane bagasse. The high contribution of distillation (91%), see Figure 3a, align with the results of Arcentales-Bastidas et al. and Oliveira and Cruz who identified utilities use at distillation as the primary source of GHG emissions in a GtG LCA.^12,18^ The higher emissions of Bolivia are remarkable, possibly because of the lack of heat integration techniques.^13^

Similar to our study, Liu et al. have highlighted the importance of using bagasse as an energy source, which substantially lowers fossil fuel inputs.^15^ However, unlike some LCAs considering the Brazilian context reporting higher emissions due to land-use change or reliance on supplementary fossil energy for distillation, Bolivia’s approach benefits from localized policies that incentivize biomass-based cogeneration.^12,16^ GtG results reported in this work also align with second-generation LCA results from an Argentinian perennial showing a competitive carbon intensity, though distinct agricultural conditions and conversion efficiencies produce slightly different emissions profiles.^27^ This comparative framework underscores the pivotal role of regional variables, such as feedstock supply and altitude-adjusted vehicle performance, in shaping the overall greenhouse gas reductions of sugar cane ethanol.

Despite the clear environmental benefits of ethanol, scaling up Bolivia production requires careful consideration. Mercado et al.^11^ evaluated Bolivia’s agricultural capacity to ensure sufficient sugar cane production without compromising food security or other sectors. Currently, Bolivia allocates 182,202 ha for sugar cane cultivation, which is projected to yield 10.2 million tons per year. Expanding sugar cane production to meet ethanol demand must be balanced with land use issues, as increasing agricultural land could potentially negate GHG emission reduction.^6,11^

### 3.3 Revenue and Profitability

The Bolivian government highlighted the significance of anhydrous ethanol plants in meeting GHG reduction targets through biofuel supply. The total capital investment value for a capacity of 216 million L per year is 199.01 million USD, see Figure S5 (Supporting Information, Section S5), is similar to the total capital investment reported by Da Luz et al.^14^ (97.6 million USD considering the Brazilian scenario and an annual production of 180.3 million L). In the same way, the total capital investment is near to those reported by Vasconcelos et al.^29^ (230 million USD for a milling capacity of 2.4 million tons per year), Kumar et al.^30^ (220.5 to 364.4 million USD for a production capacity ranging between 23.49 and 66.10 million gallons per year), Zhou et al.^31^ (188 million USD for a capacity of 4.09 × 10^7^ kg per year of ethanol), and Joseph et al.^24^ (17 USD per gallon for a capacity of 7 million gallons per year).

The annual production cost of 170 million L/y was 87.30 million USD (Figure S6), similar to those reported by other studies. Vasconcelos et al.^29^ calculated an annual production costs of 0.50 USD per liter, and Kumar et al.^30^ reported annual production costs of 0.53 USD per liter for production capacities of about 1.6 million tons per year. Oliveira and Cruz^12^ reported that sugar cane can represent between 35 and 46% of the annual production cost, and Zhou et al.^31^ reported that the raw material costs (involving sugar cane) represented 54.8% of the annual production cost, highlighting the impact of sugar cane cost on the economic assessment.

The profitability results corroborate those of Vasconcelos et al. and Kumar et al., who reported an increase in the IRR when the production capacity increases, due to the reduction in the production costs.^29,30^ The IRR of 7.93% in this study for SB2 scenario (see Table 1), is lower than the IRR reported by Joseph et al.^24^ (between 10 and 20%) this is explained by the fact that sugar cane costs for 2032–2045 period considers an annual inflation rate given the Bolivian context and an international reference sugar cane cost. The MESP considering the SB2 scenario is 0.584 USD/L (see Table 1) and becomes competitive in relation to what was reported by Oliveira and Cruz (600–660 USD/m^3^) and by Zhou et al.(0.81 USD/kg).^12,31^ This is possible for the subsidies provided by the government, resulting in a low cost of raw materials within the ethanol production chain along the distillery lifespan.

**Table 1:** Profitability Indicator for Ethanol Production in Bolivia

parameter	without expansion (SB1)	with expansion (SB2)
NFV	76.96 million USD	258.30 million USD
simple paybackTime	19 years 4 months	11 years 9 months
NPV	43.43 million USD	202.94 million USD
discounted payback time	20 years	11 years + 9 months
IRR	2.68%	7.93%
MESP	0.677 USD/L	0.584 USD/L

Government policies promote sugar cane crop expansion for ethanol production, and as result, Guabirá mill achieved a milling record of 3 million tons in 2023, necessitating 59,000 ha, with a forecasted expansion of 307,000 ha proposed by the government.^6,11^ Aspen Plus v14 calculated an annual potential anhydrous ethanol production of 216 million liter, surpassing Guabirá’s 2023 production of 108 million liter. Investments in capital and operational expenditures, such as the 2021 increase in distillation capacity, render the expansion feasible.

#### 3.3.1 Sensitivity Analysis

The sensitivities of NPV and MESP to market variation, technical uncertainties, and labor legislation were evaluated. As shown in Figure 4a, potential increases in ethanol price demonstrated a more substantial impact on NPV, such that a 10% increase could result in an NPV of 317.25 million USD (an increase of 56.3% relative to 202.94 in the base case). Given that all costs are negatively correlated with NPV, and positively correlated with MESP, the increase in equipment cost exhibits a significant impact on NPV and MESP (see Figure 4a,b); a 30% increase could lead to an 89.3% decrease in NPV (21.81 million USD) and to a 15.4% increase in MESP (0.674 USD/L). Both results support the research of Oliveira and Cruz. and Zhou et al., who reported that the NPV is especially sensitive to ethanol price and equipment investment.^12,31^

**Figure 4:**
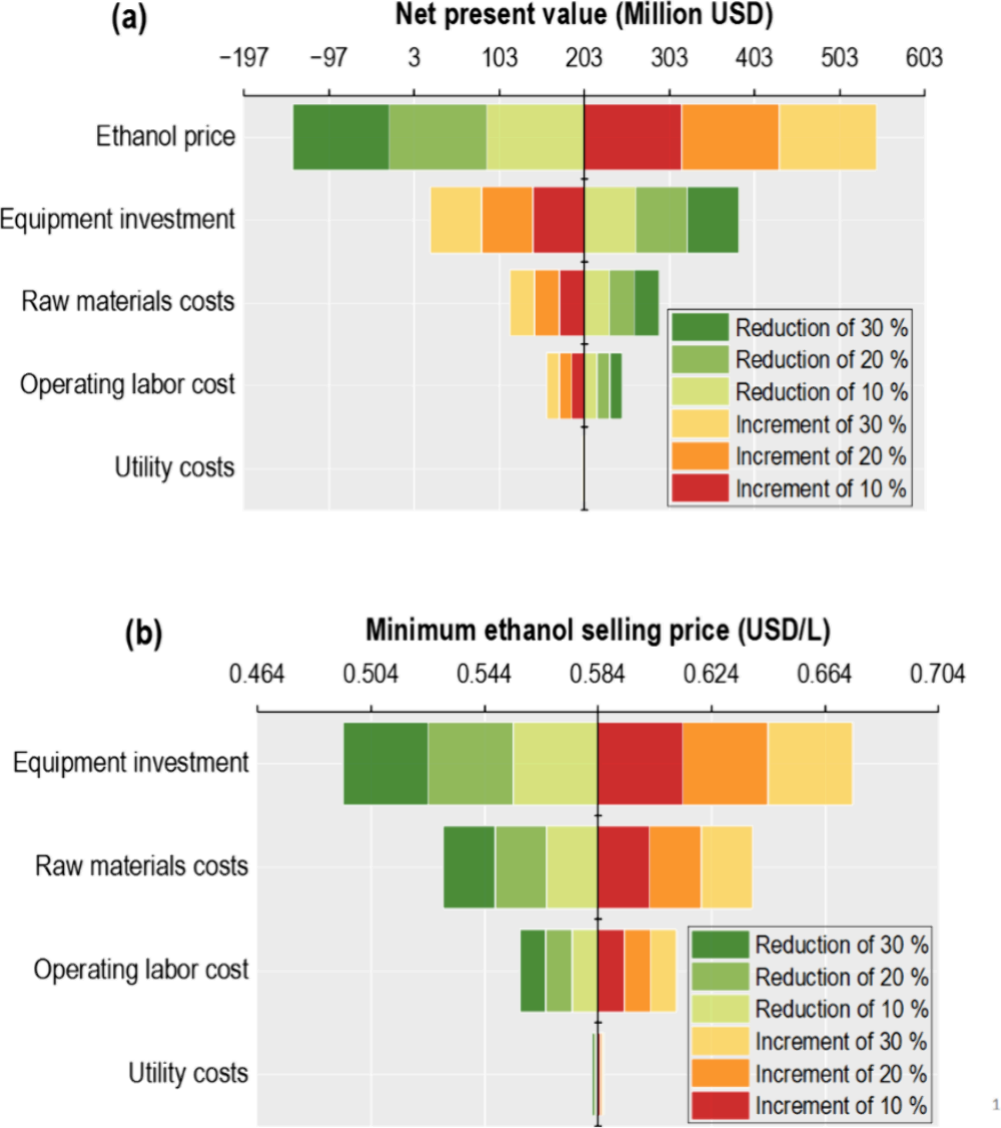
(a) Sensitivity analysis of the NPV; (b) sensitivity analysis of the MESP.

Operating labor and raw material costs have a significant impact on profitability; a 30% increase in both would reduce the NPV by 21.9 and 43.2%, respectively. Similarly, the MESP is especially sensitive to the raw material cost (see Figure 4b), which can reach up to 0.638 USD/L with an increase of 30% in this variable. Variations in utility costs have a negligible effect on the NPV and MESP. Bolivia’s economic environment supports profitable growth, and prioritizing ethanol export markets is advisible because ethanol prices have a significant impact on the NPV. The potential for electricity sales and sugar cane advantages makes Bolivia’s biorefinery industry lucrative. Government support remains essential to sustain this competitive edge amid global competition.

Bolivia shows growth potential, with Guabirá’s production reaching 108 million liters by 2023–2024 and further expansion indicating market share growth. The proposed expansion to 307,000 ha provides a strategic plan.^11^ Bolivia’s landlocked position complicates ethanol export logistics, limiting competitiveness with major producers like Brazil. The small domestic market necessitates export reliance on economies of scale, focusing on cost control and competitive pricing.

### 3.4 Bolivia’s Ethanol Scenario in Automotive Transportation

In Bolivia, the proportion of ethanol in automotive fuel increases with blend rates ranging from 12 to 25% from 2022 to 2028. Figure 3b shows that the E12 blend (the current blend in Bolivia) has the potential to reduce GHG emissions compared to pure gasoline. Passenger cars can reduce their GHG emissions from 227.96 to 214.33 gCO_2_eq/km for FFV, from 198.33 to 189.80 gCO_2_eq/km for SIDI and from 153.23 to 146.44 gCO_2_eq/km for HV. The same trend was also observed for light-duty trucks SUVs and light-duty trucks.

However, considering the highest ethanol blend (E25) of the Bolivian legislation and pure gasoline, it is possible to appreciate that the GHG emissions in passenger cars achieved reductions of 10.3% for FFV, 9.0% for SIDI, and 11.2% for HV. For light-duty trucks, emissions decreased by 12.5% for FFV, 11.1% for SIDI, and 13.2% for HV, whereas GHG emissions decreased by 15.2% for FFV, 13.9% for SIDI, and 14.7% for HV.

GHG emissions from FFV passenger cars using E12 decreased from 214.33 gCO_2_eq/km in 2022 to 204.40 gCO_2_eq/km by 2028 with E25, a 4.6% reduction (Figure 3b). The SIDI vehicle emissions decreased by 4.9%, whereas HV exhibited the most significant reduction (7.2%). For light-duty trucks SUVs, FFV emissions decreased from 190.46 gCO_2_eq/km in 2022 to 177.33 gCO_2_eq/km by 2028 (6.9% reduction) with a 25% ethanol blend. SIDI trucks showed a 7.1% reduction, and HV demonstrated a 9.3% drop. The most significant reduction in light-duty trucks was observed in HV (10.9%), followed by SIDI vehicles (10.0%) and FFVs (9.8%). These reductions are notable because Bolivia’s transportation sector significantly contributes to national GHG emissions and these three vehicle categories represent 52.4% of the national fleet.^2,9,25^

These findings align with those of Kroyan et al., who reported a TtW reduction of 5.4% in CO_2_ emissions for an E85 blend, based on fuel consumption data for ethanol blends from 5 to 60 L/100 km, indicating a smaller reduction than in the current study.^32^ However, they noted that as the ethanol fraction increased, the carbon content of the blends decreased, enhancing the energy conversion efficiency. Similarly, Santoyo-Castelazo et al. found that the TtW GWP of the E5 blend in Mexico is 2% lower than that of pure gasoline.^13^

Bolivia’s domestic supply of sugar cane ethanol is designed to supplement, rather than entirely replace, the existing fossil-based gasoline. The current blending targets (E12 and, ultimately, E25) allow ethanol to be blended at levels consistent with the available distillery capacity and fit into the existing fuel distribution network. As production expands, with planned mill upgrades and government-supported increases in sugar cane acreage, the share of ethanol in gasoline blends will grow proportionally, gradually displacing imported fuels. By aligning ethanol output with the pace of incremental blend mandates, Bolivia can ensure that its ethanol supply dovetails with conventional fuel demand, maintaining stable prices and volumes while steadily boosting the fraction of renewable content in the country’s transportation sector.

Bolivia’s projected increase in ethanol content in fuel blends (E25) is anticipated to reduce CO_2_ emissions and fossil fuel consumption, aligning with global trends such as ED95 in Thailand and E40.^22^ Canadian research on midlevel ethanol blends (E15–E30) indicates potential well-to-wheel greenhouse gas (GHG) emission reductions ranging from 7.2% (corn ethanol) to 13.4% (cellulosic ethanol) for light-duty fleets by 2030.^33^ India’s E20 blend target for transportation, aimed at reducing oil imports and emissions, demonstrates substantial reductions in spark ignition engine vehicle emissions due to lower sulfur, aromatic, and benzene contents in gasoline.^3,19^

#### 3.4.1 Air Pollutants and Tailpipe Emissions

This study indicates that higher ethanol blends (E12–E25) significantly reduce tailpipe emissions and enhance the combustion efficiency in Bolivia’s vehicle fleet. Prior research supports this, showing that the high oxygen content of ethanol improves combustion and lowers emissions of VOCs, NO*_x_*, CO, and SO*_x_* compared to conventional gasoline.^1,23^ With an increase in ethanol concentration, VOC emissions (Figure 5a) significantly decreased across the various vehicle types. Specifically, passenger cars (FFV, SIDI, and HV) saw up to a 21.0% reduction in VOC emissions with E25 compared to gasoline. Light-duty trucks and SUVs experienced a reduction of up to 20.9%, with FFVs achieving the highest reduction. These findings align with those of Shet and Moholkar, who noted decreased VOC emissions with higher ethanol concentrations owing to improved combustion efficiency (facilitated by the high oxygen content of ethanol), reduced VOC formation, and enhanced urban air quality.^1^

**Figure 5:**
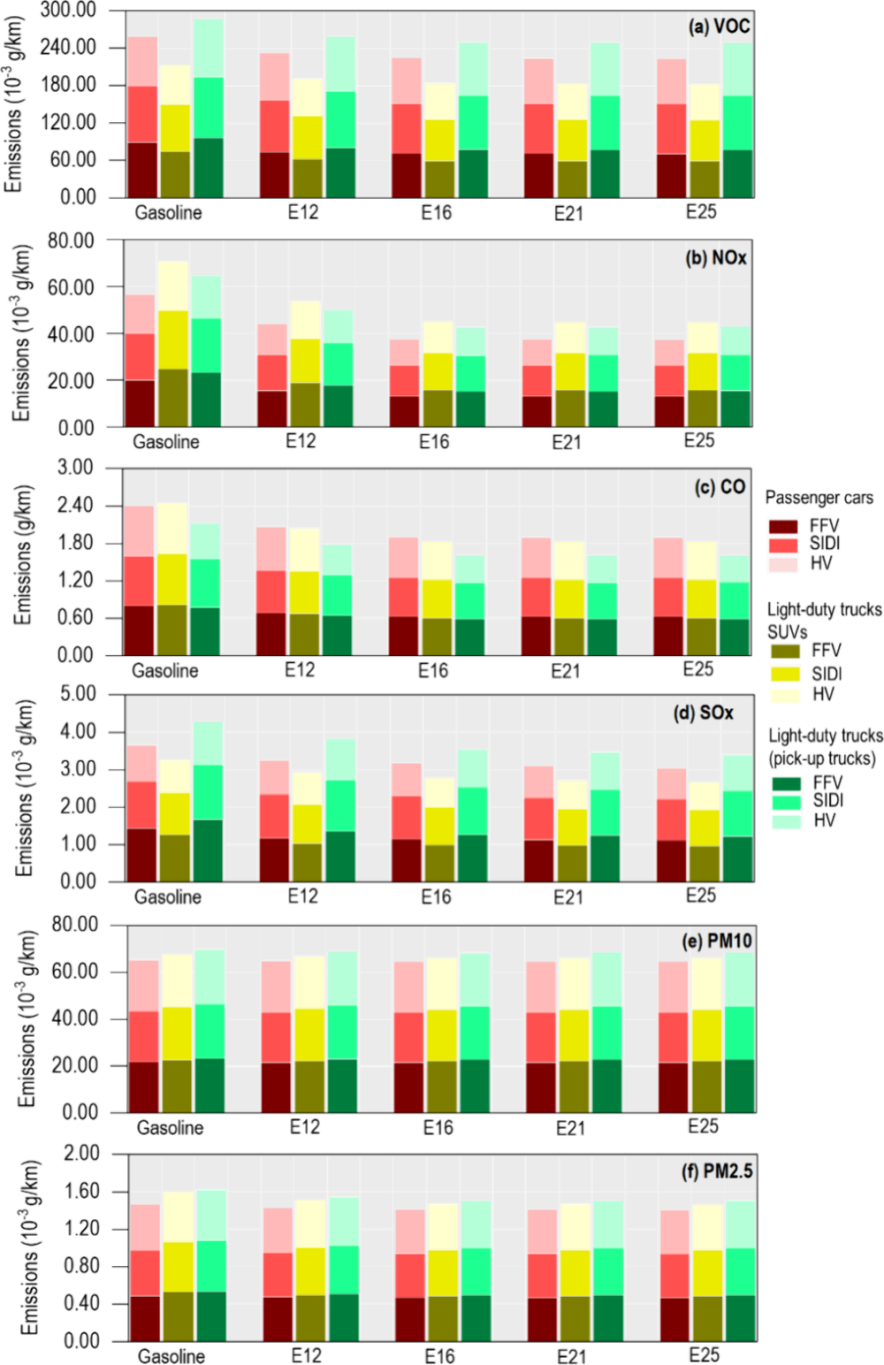
Tailpipe emissions of (a) VOC, (b) NO_*x*_, (c) CO, (d) SO*_x_*, (e) PM10, and (f) PM2.5, for different categories: passenger cars, light-duty trucks SUVs (SUVs and CUVs), and light-duty trucks (only pick-up trucks). The vehicle technologies considered include flexible fuel vehicles (FFV), spark ignition and direct injection (SIDI), and hybrid vehicles (HV).

Ethanol blending also significantly reduced the NO_*x*_ emissions (Figure 5b). In passenger cars, the reduction in NO*_x_* emissions dropped 15.2%, with an increase in ethanol content from E12 to E25. Similar reductions were observed for the light-duty trucks (16.3%) and SUVs (14.3%). These results are consistent with the findings of Ye et al., who reported a decrease in NO*_x_* emissions with higher ethanol blends in SIDI engines.^34^ García et al. highlighted the advantages of oxygenated fuels such as ethanol in reducing NO*_x_* emissions without the need for major vehicle modifications, suggesting that the integration of ethanol-blended fuels could be an effective approach for mitigating urban air pollution in Bolivia.^21^ Nitrogen oxides contribute to smog formation and respiratory issues, making their reduction crucial in improving public health in densely populated areas.

Carbon monoxide (CO) emissions (Figure 5c) were also reduced with increasing ethanol content, with reductions ranging from 21.2% in passenger cars to 25.1% in light-duty trucks and SUVs when comparing E25 with gasoline. This reduction in CO emissions is attributed to the ability of ethanol to improve combustion efficiency, thereby reducing the formation of partially oxidized products such as CO. Jamrozik and Tutak and García et al. emphasized the role of oxygenated fuels in converting CO to less harmful CO_2_.^5,21^ This reduction is particularly significant in Bolivia, where CO is a major contributor to air pollution and respiratory problems, supporting Bolivia’s objective of improving the air quality and protecting public health. Moreover, Altitude negatively affects complete combustion and increases CO emissions; thus, the presence of oxygenated chemicals in fuel blends, such as ethanol, can have an important impact on air quality in urban centers.

The study also showed that sulfur oxides (SO*_x_*) emissions (Figure 5d) decreased with higher ethanol blends. Although the reduction in SO*_x_* was modest, it still represented a meaningful improvement in air quality. For example, in passenger cars, SO*_x_* emissions decreased by 8.8%, whereas light-duty trucks and SUVs showed reductions of up to 12.4%. This reduction is consistent with studies suggesting that the lower sulfur content in ethanol-blended fuels contributes to the reduced SO*_x_* emissions from combustion.^35^ Given Bolivia’s high reliance on fossil fuels and its associated SO*_x_* emissions, transitioning to ethanol blends offers a promising solution for reducing these pollutants and improving urban air quality.

Although ethanol blending proved effective in reducing VOCs, NO*_x_*, CO, and SO*_x_* emissions, the reduction in particulate matter (PM10 and PM2.5) was minimal (Figure 5e,f). This is in line with studies by Hutchison and Wallace, who found no significant changes in PM emissions with increasing ethanol blends in SIDI engines.^36^ Therefore, addressing particulate matter pollution in Bolivia will require complementary measures, such as the implementation of advanced emission control technologies in vehicles and the adoption of ethanol-blended fuels.

Considering Bolivia’s objective to mitigate its environmental impact while managing economic growth, the implementation of ethanol-blended fuels (E12–E25) could constitute a viable approach for attenuating VOC emissions while augmenting the local fuel production capacity.^8,9^ However, to address PM pollution, additional strategies must be implemented along with ethanol blending. Prioritizing ethanol use in high-traffic areas and expanding the distribution infrastructure could further enhance air quality, especially in urban regions with limited air quality control.^37^ The use of ethanol blends represents a viable and sustainable solution for Bolivia in reducing tailpipe emissions, supporting energy security, and improving public health, particularly in vulnerable communities.^23^

### 3.5 Global Comparison and Perspectives

Global ethanol use in transportation is increasing with significant integration efforts by different countries. In Brazil, gasoline contains 18–27% ethanol, and its successful ethanol program exemplifies the effective use of high-ethanol blends in FFV and traditional gasoline vehicles, reducing fossil fuel imports and carbon emissions.^16,17^ India mandated a 20% ethanol blend (E20) by 2025 to cut oil imports and address rising energy demand, highlighting the use of ethanol as a key energy source.^3,4^ Bolivia also stands to gain from higher ethanol blends because of its potential for sugar cane and other biobased ethanol production.^2,9^ The global ethanol push supports GHG emission reductions in line with the Paris Agreement and other climate targets, with ethanol cutting CO_2_ emissions by 20–30% compared to conventional gasoline, which is crucial for a low-carbon transition.

Comparative studies show Bolivia’s ambitious but achievable targets, with economic and social benefits from domestic ethanol production and rural development. By 2028, employing FFV, SIDI, and HV with up to 25% ethanol blends could reduce CO_2_ emissions, highlighting the role of ethanol in lowering transportation emissions. Nonetheless, ethanol blends in Bolivia did not reduce particulate matter (PM) emissions, as observed in developed countries,^1,35^ likely because of older vehicle fleets and less advanced emission control technologies.

Although first-generation sugar cane ethanol remains predominant in many South American contexts, the convergence of second-generation fermentation, coproduct valorization, and improved steam economy continues to shape the technological landscape. Studies in Brazil and Argentina have demonstrated how 2G fermentation, combined heat and power (CHP) systems, and enhanced distillation techniques can significantly lower the carbon intensity of sugar cane ethanol while improving economic margins.^15,24,27^

Aligning ethanol production with environmental goals is vital for the success of Bolivia. Further optimization of ethanol production in Bolivia could be achieved by integrating technological innovations and supportive policies tailored to local conditions. One potential improvement is advanced heat integration strategies in the distillation and dehydration stages, such as vapor recompression or multieffect evaporation, significantly reducing steam demand and lowering the life cycle greenhouse gas (GHG) emissions by cutting fossil backup needs.^12,13^ Similarly, implementing second-generation fermentation technologies that use sugar cane residues (like bagasse or filter cake) would allow more efficient feedstock utilization and potentially increase total ethanol yields without expanding agricultural land.^9^ From a policy standpoint, incentivizing investments in cutting-edge cogeneration systems, carbon capture at industrial emission points, and precision agriculture could strengthen the economic viability of these technologies.^8,9^ Additionally, performance-based subsidies, geared toward demonstrated emissions reductions or the adoption of novel process improvements, would help align producers’ incentives with Bolivia’s national decarbonization targets. By combining these technological and policy measures, Bolivia could not only refine its existing ethanol production processes but also position local sugar cane mills (as Guabirá, Unagro, Aguaí and La Bélgica) as a regional leader in sustainable biofuel development.^11^

## 4 Conclusions

The findings of this study underscore Bolivia’s potential to leverage sugar cane ethanol as a sustainable solution for reducing transportation sector emissions and enhancing energy security. With a greenhouse gas emissions profile of 1.61 gCO_2_eq/MJ, significantly lower than gasoline’s 70.74 gCO_2_eq/MJ, Bolivia’s ethanol production presents a viable pathway to decarbonization. The projected implementation of ethanol-gasoline blends (E12–E25) could reduce vehicular emissions by up to 15.2% in pick-up trucks and 11.2% in passenger cars by 2028, while also lowering carbon monoxide and volatile organic compound emissions by 25.1 and 21.0%, respectively. Economically, expanding ethanol production to 170 million liters annually yields a net present value of 202.94 million USD and an internal rate of return of 7.93%, reinforcing its profitability. These results highlight Bolivia’s strategic opportunity to transition toward a low-carbon transport system, aligning with global sustainability targets, and contributing to climate change mitigation through biofuel integration.

## Supplementary Material





## Notes

The authors declare that the research data supporting this publication are available from the repository at https://domusdados.unifesp.br/dataverse/ggesepb. (Section 1) Thermodynamic model; (Section S2) process simulation description; (Section S3) life-cycle assessment; (Section S4) assumptions for the economic evaluation; (Section S5); and additional information on the economic evaluation (PDF)
Data set of the road transport fleet in Bolivia (XLSX)
