# Supplementary material for: Bolivia’s Path to Lower Emissions: Sugar Cane Ethanol and GREET Model Insights into Ethanol-Gasoline Blends
Source: ACS Omega. 2025 Jun 12;10(24):25350–61. doi: 10.1021/acsomega.5c00346 (PMC12199017; doi:10.1021/acsomega.5c00346)
Supplement: Supplementary file 1 [file ao5c00346_si_001.pdf]

Supporting Information

Bolivia's Path to Lower Emissions: Sugarcane Ethanol and GREET Model Insights on Ethanol-Gasoline Blends

Victor S. V. Mercado[†]*, Romilda Fernandez-Felisbino*[†]*, Jean F. Leal Silva*[‡]*, Laura Plazas Tovar*[‡,*]

[†]Department of Chemical Engineering, Federal University of São Paulo, Rua Prof. Artur Riedel, 275 - Eldorado, Diadema, SP, ZIP code 09972-270, Brazil

[‡] School of Chemical Engineering, University of Campinas, Avenida Albert Einstein 500 – Cidade Universitária, Campinas, SP, ZIP code 13083-852, Brazil.

[*]*To whom correspondence should be addressed: e-mail: laura.tovar@unifesp.br*

The supporting information contains the following information

Number of pages: 20 pages

Number of tables: 10

Number of figures: 6

SUMARY

Section S1……………………………………………………………….…...S3

Table S1……………………………………………………………………..S3

Table S2……………………………………………………………………..S4

Section S2...……………………………………………………………………S4

Table S3……………………………………………………………………...S7

Table S4……………………………………………………………………...S7

Table S5……………………………………………………………………...S8

Table S6……………………………………………………………………...S9

Table S7……………………………………………………………………...S9

Figure S1……………………………………………………………………..S10

Figure S2……………………………………………………………………..S11

Figure S3……………………………………………………………………..S12

Figure S4……………………………………………………………………..S13

Section S3...…………………………………………………………………..S14

Table S8………………………………………………………………………S14

Section S4...…………………………………………………………………...S15

Table S9………………………………………………………………………S15

Table S10……………………………………………………………………...S16

Section S5...…………………………………………………………………...S17

Figure S5……………………………………………………………………....S18

Figure S6……………………………………………………………………....S18

References……………………………………………………………………..S19

Section S1: Thermodynamic model

Sugarcane feedstock consisted of both conventional and hypothetical components (**Table S1**). In this study, lignocellulosic components, such as cellulose and lignin, were treated as solids using the SOLID model in Aspen Plus v14, whereas yeast properties were calculated using experimentally derived values from POPOVIC (**Table S2**).[1,2] The models used for the complete simulation were a non-random two-liquid model (NRTL-RK) to deal with the phase equilibrium of strongly non-ideal solutions, UNIQUAC during the evaporation stage (S3), as Da Luz et al. and Gong et al.[2,3] reported experimental coefficients for the binary interaction parameters of water and sucrose. Consequently, the NRTL model was used for sugarcane treatment (S1), fermentation (S2), distillation (S4), dehydration (S5), and cogeneration (S6) stages, whereas UNIQUAC was applied at the evaporation stage (S3).[4–6]

Table S1. Sugarcane composition [a]

**Compound**	**Type of compound**	**Composition (%wt)**
Water	Conventional	68.801
Sucrose	Conventional	13.212
Glucose	Conventional	0.566
Phosphoric acid	Conventional	0.0286
Minerals	Conventional (hypothetical)	0.187
Salt	Conventional (hypothetical)	1.536
Soil	Conventional (hypothetical)	1.360
Cellulose	Solid (hypothetical)	6.614
Hemi cellulose	Solid (hypothetical)	3.685
Lignin	Solid (hypothetical)	3.632

[a] The sugarcane stream was treated as a MIXCISLD class flow, accounting for both solid and liquid components

Table S2. Hypothetical compound properties

Compound	Molar mass	Molar formula	Density (kg/m^3)	Reference
Cellulose	162.14	$C_6H_{10}O_5$	1530	Oliveira and Cruz[5]
Hemi cellulose	132.12	$C_5H_8O_4$	1530	Joseph et al.[7]
Lignin	172.60	$C_9H_{10.2}O_{3.4}$	1500	Le et al.[8]
Yeast	30.23	$CH_{1.8}O_{0.9}N_{0.145}$	1000	Da luz et al.[3]

Section S2: Process simulation

(S1) Treatment. Sugarcane treatment entails cleaning, extracting, and physically processing the juice. Initially, sugarcane was cleaned to remove 70% of debris, soil, and minerals using a *component separator* model, then mixed with hot water (28% of the product stream) and processed through a second *component separator model* to remove 20% solids.[5,7] The juice was then extracted (simulated as a *component separator* model) to remove 98% of bagasse, which was then sent to a boiler for electricity generation. After extraction, the juice was heated to 70 °C to react with phosphoric acid and calcium hydroxide for flocculation (**Table S3)** using an *RStoic* model.[9] The stream was further heated to 105 °C and sent to a separator (*flash* model) to release volatile gases, and then to a decanter (*component separator* model) to remove 100% solids, 17% sucrose, and glucose.[3,10] To recover the sugars carried, the solid fraction was mixed with water at a flow rate 1.5 times greater and then directed to a second decanter, where the liquid fraction was blended with the main juice stream and directed to the next stage.

(S2) Evaporation. Part of the juice was concentrated and heated using a five-effect evaporator system, with each effect operating at different pressures and temperatures, from a temperature of 115.6 °C and a pressure of 1.7 bar in the first stage to 60.4 °C and 0.2 bar in the fifth stage (**Table S4)**, increasing the juice's Brix to 65°.[10,11] The combination of Aspen Plus v14 models (*flash*, *valve,* and *HeatX*) is illustrated in **Figure S1** and was used to simulate the evaporators. The concentrated juice was then mixed with the remaining fraction to obtain a juice of 20° Brix for fermentation.[3,12]

(S3) Fermentation. The concentrated juice was sterilized at 130 °C and 6 bar and then cooled to 32 °C for optimal fermentation. An *RStoic* model at 32 °C and atmospheric pressure was used to simulate the fermentation reactions (**Table S5)** that convert sucrose to glucose and fructose,

followed by the conversion of glucose to ethanol. Simulated byproducts of yeast metabolism included glycerol, succinic acid, acetic acid, and isoamyl alcohol. Carbon dioxide produced during fermentation was removed from the reactor outlet stream (*Flash2* model), and passed through a ten-stage absorber column (*RadFrac* model) to recover ethanol. A water mass proportion of 0.15 times the carbon dioxide feed were considered.[3,7]

The liquid stream from the fermenter was centrifuged in a *CFuge* model to separate the yeast, which was then directed to a second *CFuge* that guaranteed ethanol recovery and recirculated the solid fraction (yeast) to the fermenter. The first *CFuge* liquid outlet was mixed with the liquid outlet from the second *CFuge* model and was sent to the distillation stage. The reactor (*RStoic* model) is fed constantly with a fraction of the yeast from the second *CFuge* called pé-de-cuba, and the process is continuously fed by ammonia and sulfuric acid streams (**Figure S2).**

(S4) Distillation: The wine stream from the fermentation process has an alcohol content of 7 %wt and is heated to 93 °C. The objective of this stage was to obtain hydrous ethanol with a minimal mass fraction of 90%. The *RadFrac* model of Aspen Plus v14 was used for all columns because it is a rigorous model for simulating all types of multistage vapor-liquid fractionation operations.[13]

Wine feeds column A1 (**Figure S3)** and the vapor generated through the top feed column D, whereas column A is fed by the bottom product. The number of stages and operating pressures are listed in **Table S5**. As columns B and B1 have the same diameter, both were considered the same column in the simulation.[5] In column A1, the feed entered the top stage, the bottom product of the column (BOT-A1) had an alcohol content of approximately 0.02 %wt and is directed towards Column A. The top outlet stream of column A (40 %wt of ethanol) goes to column B-B1.[11,12]

The top liquid outlet stream of column D is divided into two sub-streams: one is recycled to the column (REC), and the other is retired as a second-class alcohol (ETH-SEG). The top gas outlet of column D (GAS-D) was composed of 90 % mass fraction of carbon dioxide.[5] The condenser was designed to operate at 35 °C and at the bottom column liquid phlegm, which is part of the feed to Column B-B1.

The top-stage stream of column B-B1 (ETH-HYD) was condensed, resulting in a vapor stream

with approximately 90% alcohol content (hydrous ethanol), whereas the liquid fraction was recirculated to the column. The Oleo fusel (OF1) contains a mixture of superior alcohols, water, and other compounds. The bottom outlet stream (PLEGHM) had an alcohol content of less than 0.002 %wt, and the column reboiler was set to achieve a temperature of 115 °C.

(S5) Dehydration: The dehydration method selected for this study was ethanol dehydration using monoethylene glycol (MEG) as the stripping component (**Figure S4**). The hydrous ethanol from the previous stage goes to the *HeatX* model (H-DEH), reaching a temperature higher than 100 °C and a pressure of 1.02 bar.[3] The stream feeds an extraction column (*RadFrac* model), where it contacts MEG, which alters the azeotropic condition and directs water to the column's bottom, while the dehydrated ethanol stream (99.8 %wt) exits from the top (anhydrous ethanol) at a temperature of 77.8 ºC. The mixture of MEG and water is directed to the recovery column (*RadFrac* model), at 158 °C and 0.3 bar, producing a water stream at the top and recovering MEG with over 98% purity at the bottom. The bottom stream was pumped, mixed with makeup, and returned to the extractive distillation column. All the column conditions are listed in **Table S6.**

(S6) Cogeneration: For Cogeneration, it is assumed that all sugarcane bagasse is used for electricity and medium- and high-pressure steam is used to supply the mill requirements. The information used in the simulation is listed in **Table S7.**

Table S3. Chemical reactions during the whole process

Description	Reaction	Conversion
Calcium phosphate formation	$3\ Ca(OH)_2 + 2\ H_3PO_4 \rightarrow Ca_3(PO4)_2$ (solid) $+ 6\ H_2O$	100 % of calcium hydroxide
Conversion of sucrose to glucose	$C_{12}H_{22}O_{11} + H_2O \rightarrow 2\ C_6H_{12}O_6$	100% of sucrose
Ethanol production	$C_6H_{12}O_6 \rightarrow 2\ C_2H_5OH + 2\ CO_2$	91% of glucose
Glycerol formation	$C_6H_{12}O_6 + 4\ H^+ + 4\ e^- \rightarrow 2\ C_3H_8O_3$	2.58% of glucose
Succinic acid formation	$C_6H_{12}O_6 + 2\ H_2O \rightarrow C_4H_6O_4 + 2\ CO_2 + 5\ H_2$	0.29% of glucose
Acetic acid formation	$C_6H_{12}O_6 + 2\ H_2O \rightarrow 2\ C_2H_4O_2 + 2\ CO_2 + 4\ H_2$	1.15% of glucose
Isoamyl alcohol formation	$C_6H_{12}O_6 \rightarrow 0.795\ C_5H_{12}O + 2.025\ CO_2 + 1.155\ H_2O + 0.075\ H_2 + 0.15\ H^+ + 8\ e^-$	0.03% of glucose
Yeast formation	$0.1485\ C_6H_{12}O_6 + 0.145\ NH_3 + 0.1087\ CO_2 \rightarrow CH_{1.8}O_{0.9}N_{0.145} + 0.2087\ H_2O$	100% of ammonia
Combustion of cellulose	$C_6H_{10}O_5 + 6\ O_2 \rightarrow 6\ CO_2 + 5\ H_2O$	98% of cellulose
Combustion of hemicellulose	$C_5H_8O_4 + 5\ O_2 \rightarrow 5\ CO_2 + 4\ H_2O$	98% of hemicellulose
Combustion of lignin	$C_9H_{10.2}O_{3.4} + 9.85\ O_2 \rightarrow 9\ CO_2 + 5.1\ H_2O$	98% of lignin

Table S4. Evaporation stage operating conditions

Effect	Pressure (bar)	Temperature (ºC)
First	1.7	115.6
Second	1.4	109.8
Third	1.0	100.3
Fourth	0.54	84.3

Table S5. Distillation stage operating conditions

**Column A1**		**Reference**
Number of stages	8	Da Luz et al [3]
Top stage pressure (bar)	1.36	Da Luz et al [3]
Bottom stage pressure (bar)	1.39	Da Luz et al [3]
Feed stage	1	Santoyo-Castelazo et al [6]
**Column A**		
Number of stages	19	Oliveira and Cruz [5]
Top stage pressure (bar)	1.39 bar	Kumar et al.[14]
Bottom stage pressure (bar)	1.53 bar	Kumar et al.[14]
Feed stage	1	Kumar et al.[14]
**Column D**		
Number of stages	6	Albarelli et al. [12]
Top stage pressure (bar)	1.35 bar	Albarelli et al.[11]
Bottom stage pressure (bar)	1.38 bar	Da Luz et al. [3]
Feed stage	6	Da Luz et al. [3]
Condenser temperature	35 ºC	Joseph et al.[7]
**Column B-B1**		
Number of stages	46	Albarelli et al. [12]
Top stage pressure (bar)	1.16 bar	Albarelli et al. [12]
Bottom stage pressure (bar)	1.32 bar	Albarelli et al. [12]
Feed stage	21	Albarelli et al. [12]
Condenser temperature (ºC)	35 ºC	Albarelli et al. [12]
Reflux ratio (mass base)	36.2	Albarelli et al. [12]
Side stream flowrate (OF1) (kg/h)	162 kg/h (stage 21)	Albarelli et al. [12]
Side stream flowrate (OF2) (kg/h)	24 (stage 45)	Albarelli et al. [12]

Table S6. Dehydration stage operating conditions

**Column of Extraction (COL-EXT)**		**Reference**
Number of stages	24	Da Luz et al [3]
Top stage pressure (bar)	1.01	Da Luz et al [3]
Bottom stage pressure (bar)	1.02 bar	Da Luz et al [3]
Solvent feed stage (bar)	24	Da Luz et al [3]
Hydrated ethanol feed stage (bar)	5	Da Luz et al [3]
Reflux ratio (mass base)	0.3	Da Luz et al [3]
**Column of Extraction (COL-EXT)**		
Number of stages	10	Morais et al [10]
Top stage pressure (bar)	0.20 bar	Morais et al [10]
Bottom stage pressure (bar)	0.30 bar	Morais et al [10]
Feed stage	5	Morais et al [10]
Reflux ratio (mass base)	0.25	Morais et al [10]
Reboiler duty (kW)	10	Morais et al [10]

Table S7. Cogeneration stage operating conditions

Cogeneration inputs		Reference
Number of steam expansion turbines	3	Sinnott and Towler[15]
Operation vapor pressure (bar)	65	Albarelli et al.[12]
Operation temperature used in the simulation (ºC)	450	Morais et al,[10]
Turbine 1 outlet pressure (bar)	10	Oliveira and Cruz. [5]
Isentropic efficiency (%)	85%	Albarelli et al.[12]
Turbine 2 outlet pressure (bar)	5	Oliveira and Cruz. [5]
Isentropic efficiency (%)	82%	Albarelli et al.[12]
Turbine 3 outlet pressure (bar)	0.22	Oliveira and Cruz. [5]
Isentropic efficiency (%)	80%	Albarelli et al.[12]

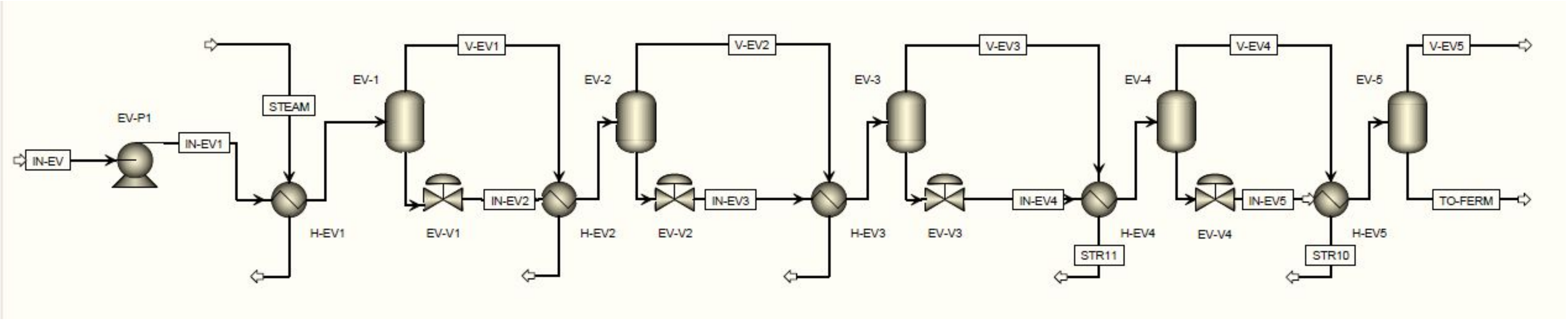

Figure S1. Flowsheet of a five-effect evaporator, combining *the flash*, *valve,* and *HeatX* models in Aspen Plus v14

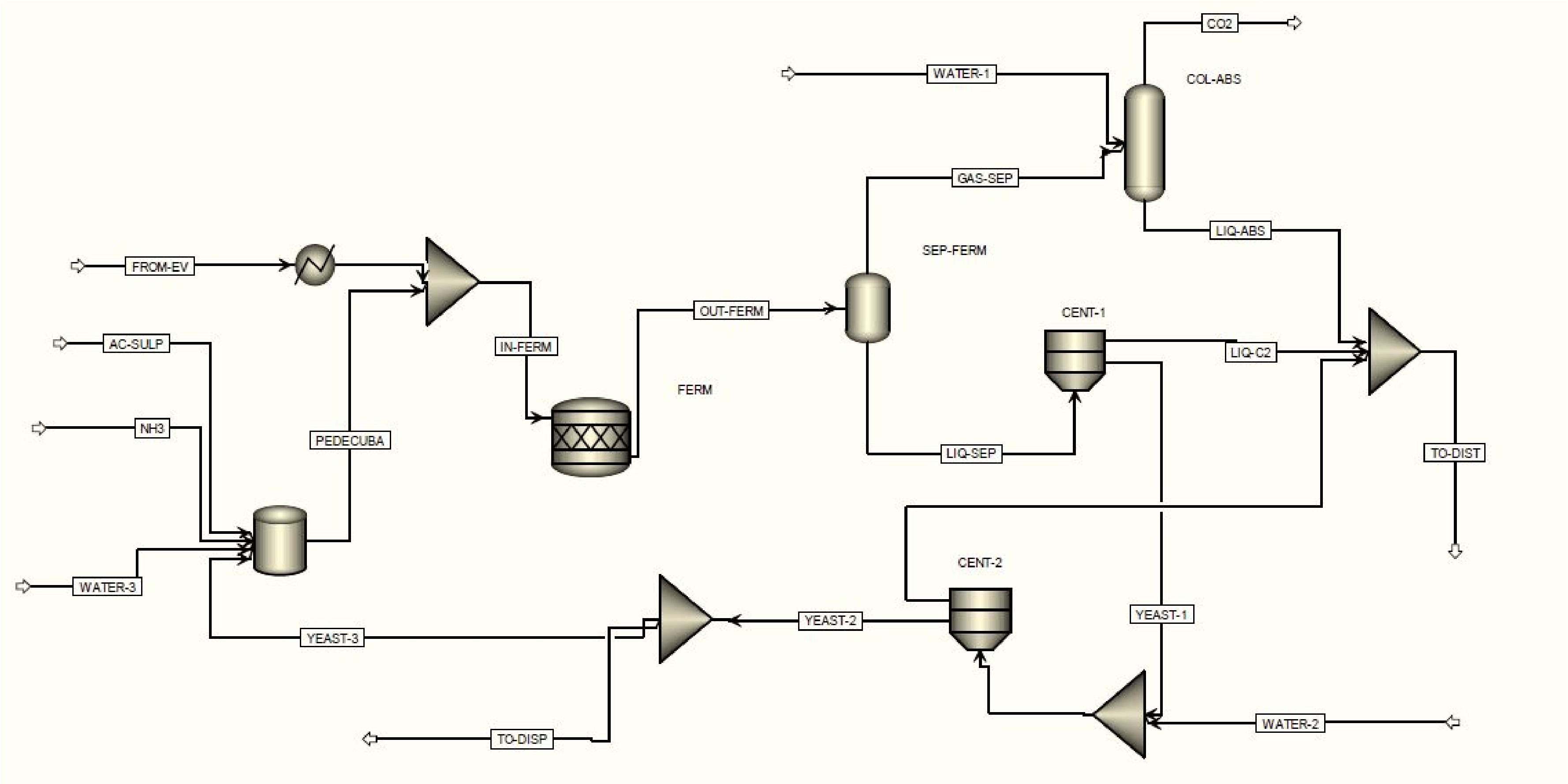

Figure S2. Flowsheet of fermentation stage in Aspen Plus v14

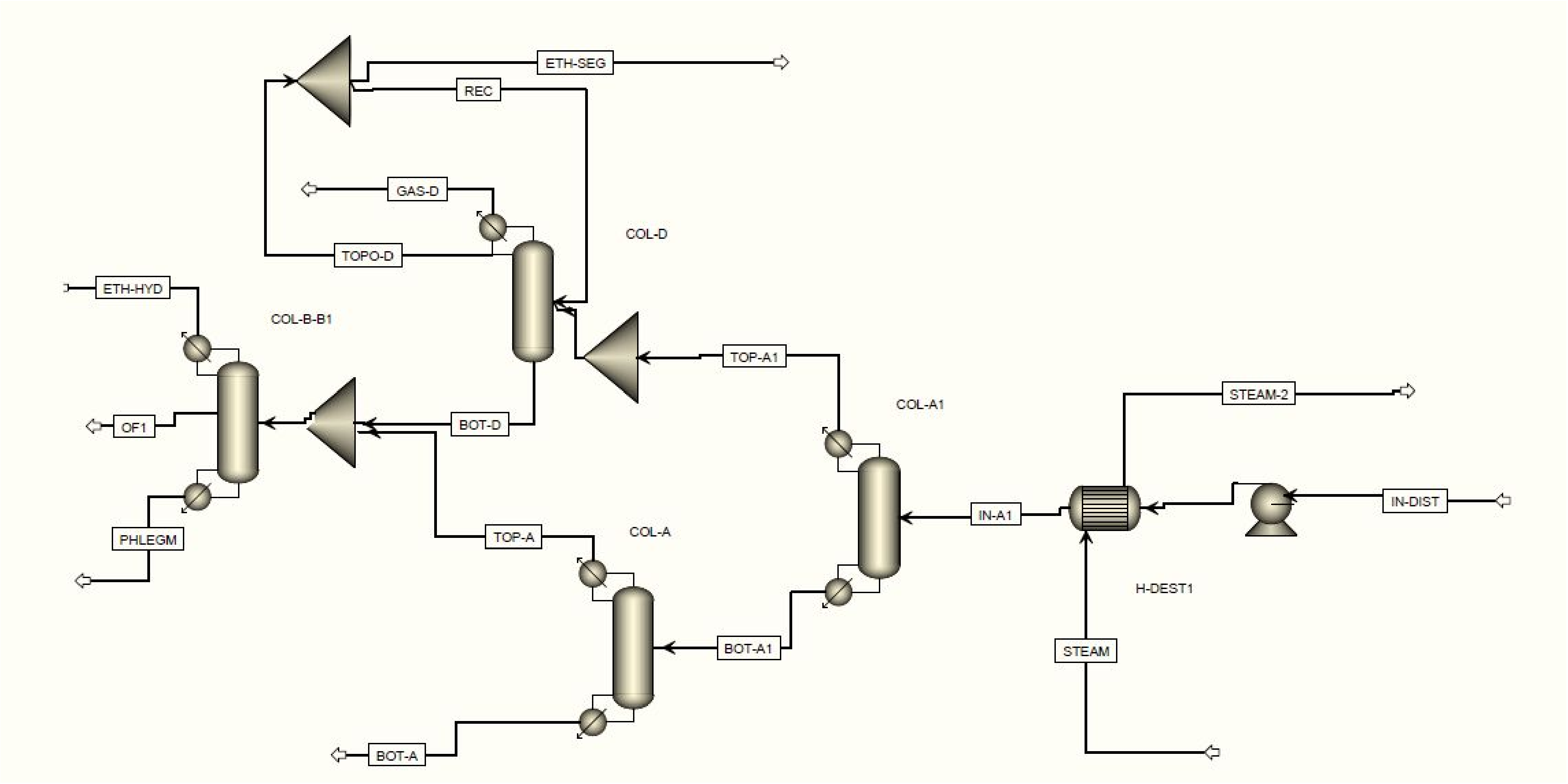

Figure S3. Flowsheet of distillation stage in Aspen Plus v14

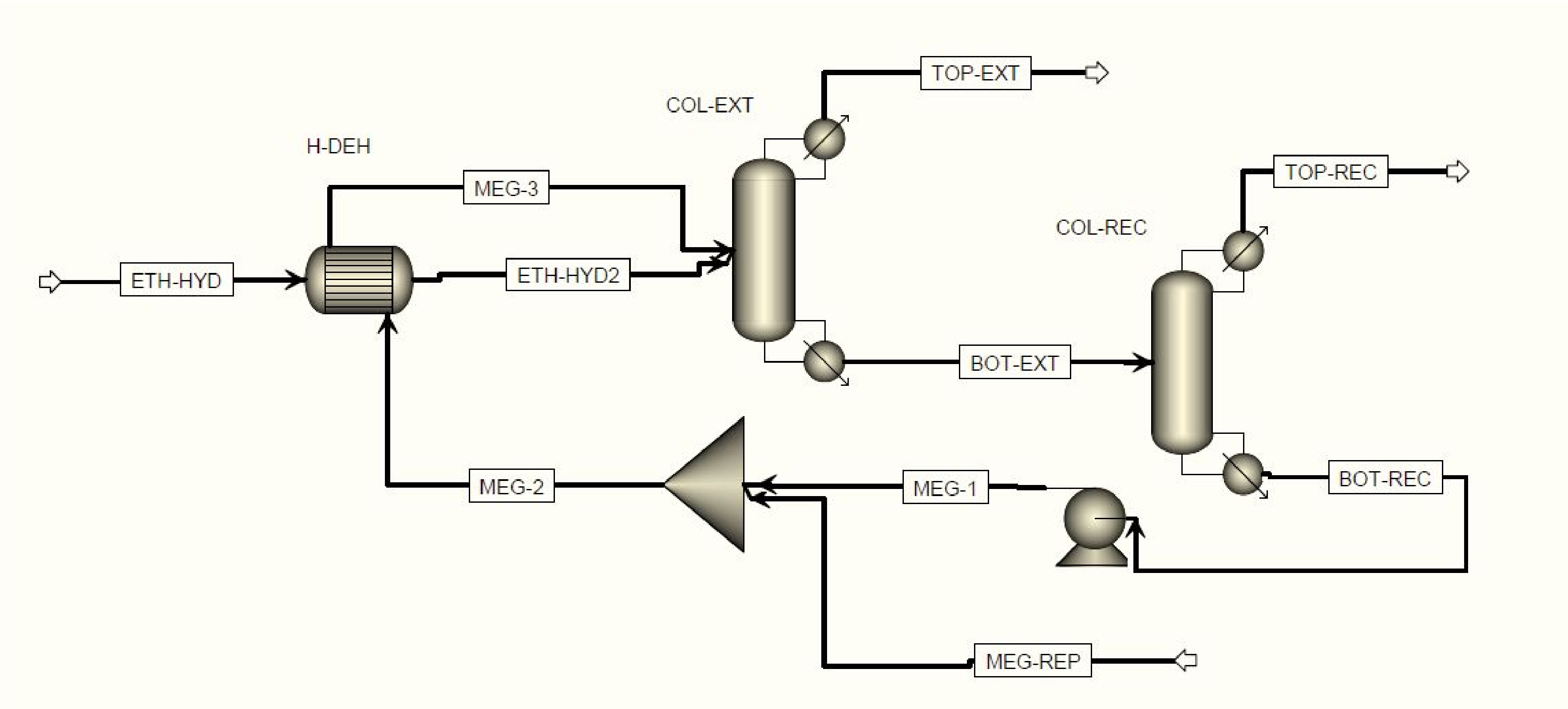

Figure S4. Flowsheet of dehydration stage in Aspen Plus v14

Section S3: Life-cycle assessment

The case study of the Guabirá distillery in Bolivia yielded 61 m³/h of ethanol, the life cycle inventory obtained through mass balances in Aspen Plus v14 simulation environment for such production capacity is detailed in **Table S8**.

Table S8. Summary of Life cycle inventory information

Parameter	Value	Reference
Milling capacity	23000 t/d	MDPyEP.[16]
Type of distillery	Annexed distillery [a]	MDPyEP.[16]
Treatment process		
Phosphoric acid	0.175 kg/t of sugarcane	Simulation results
Water (50 °C)	339.58 kg/t of sugarcane	Simulation results
Calcium hydroxide	1.980 kg/t of sugarcane	Simulation results
Fermentation process		
Sulfuric acid	0.00867 kg/t of sugarcane	Simulation results
Water (25 °C)	2.296 kg/t of sugarcane	Simulation results
Ammonia	0.315 kg/t of sugarcane	Simulation results
Water (30 °C)	53.051 kg/t of sugarcane	Simulation results
Dehydration process		
Mono ethylene glycol	584.169 kg/m^3 of ethanol	Simulation results

[a] The facility under investigation is an annexed distillery plant with a sugarcane mill integrated into the process. However, sugar production was not included in this study because sugar production in Bolivia has been operational for over three decades. This information was verified through historical records of the Guabirá distillery in Santa Cruz, Bolivia, and the broader Bolivian context.

Section S4: Assumptions for the economic evaluation

Based on 2021-2022 Bolivian contract trends, two scenarios were analyzed for economic evaluation: SB1, without production expansion, and SB2, considering a production expansion. The number of harvest days per season and the ethanol price of the period 2024-2025 where considered for each scenario, see **Table S9.** Additionally, all the costs involved in the total capital investment calculation, as well as those involved in the annual production cost where estimated using appropriate multiplication factors and available data of the raw materials supply, see **Table S10.**

Table S9. Scenarios for ethanol production expansion

Period	Ethanol production million L/year		Ethanol price USD/year	Number of harvest days per season
	SB1	SB2		
2019-2020	0	0	0.609	148
2021-2022	40	40	0.710	148
2022-2023	70	70	0.710	148
2025-2031	100	130	0.710	200
2032-2038	130	170	0.710	200
2039-2045	150	170	0.710	200

Table S10. Cost assumptions and information for the economic evaluation

Input	Cost	Reference
Raw materials		
Sugarcane	Not considered from 2021 to 2032	
Sugarcane	26.43 $/t (2033–2045)	Oliveira and Cruz.[5]
Phosphoric acid	420 $/t	Kumar et al.[14]
Calcium hydroxide	410 $/t	Kumar et al.[14]
Sulfuric acid	0.1 $/kg	Moncada et al.[17]
Water (25 °C)	1.97×10^{-5} $/kg	MDPyEP.[16]
Ammonia	0.42 $/kg	Company reports.[a]
Water (30 °C)	1.97×10^{-5} $/kg	MDPyEP.[16]
Mono ethylene glycol	522 $/t	Company reports.[a]
Utilities		
Cooling water	1.97×10^{-5} $/kg	MDPyEP.[16]
Fixed cost		
Operating Labor cost	15% of APC	Sinnott and Towler.[15]
Supervision cost	7.5% of APC	Sinnott and Towler.[15]
Direct Overhead	16% of APC	Sinnott and Towler.[15]
Maintenance cost	3% of EI	Sinnott and Towler.[15]
Land and Local Property	2.6% of EI	Sinnott and Towler.[15]
Plant overheads	2.8% of APC	Sinnott and Towler.[15]
License Fees and Royalties	1% of FCI	Sinnott and Towler.[15]
Capital charges	10% of FCI	Sinnott and Towler.[15]
Insurance	1% of FCI	Sinnott and Towler.[15]
Total capital investment (TCI)		
System and facilities investment (SFI)	30% of EI	
Direct capital investment (DCI)	SFI + EI	
Indirect capital investment (ICI)	40% of DCI	
Fixed capital investment (FCI)	ICI + DCI	
Working capital (WC)	10% of TCI	
Total start-up investment (TSI)	10% of FCI	
Total capital investment (TCI)	FCI + WC + TSI	
Distribution of TCI	10% the 1st year<br>40 % the 2nd year<br>40% the 3rd year<br>10% the 4th year	

APC: Annual production cost

[a] This information was verified through historical records of the Guabirá distillery in Santa Cruz, Bolivia, and the broader Bolivian context.

Section S5: Results of the economic evaluation

Ethanol production contracts rose from 40 million liters in 2020 to 130 million liters by 2024, with planned volume growth to 2024-2045 (**Table S9**) to foster investment in sugarcane-based products. According to **Figure S5**, the equipment investment was 89.47 million USD, the systems and facilities investment was 26.84 million USD, and the indirect capital investment was 46.52 million USD, totaling 199.01 million USD.

Equipment investment is the largest component of the total capital investment, followed by indirect capital, systems, and facility investments. Evaporation accounted for 27.4%, and cogeneration systems accounted for 6.0%, owing to the extensive equipment used in these stages (see **Figure S6**). These findings align with those of other South American ethanol production studies, where reactors, vessels, cogeneration equipment, and heat exchangers constituted 65.6% of the equipment investment, increasing to 72.6% for second-generation ethanol production. Plant construction costs represent 20%-30% of the fixed capital investment. Working capital was allocated for the expenses of plant initialization (including equipment installation, buildings, instrumentation, yard improvement, and testing) and was estimated to be 10% of the total capital investment. It was calculated using appropriate multiplication factors for the equipment cost.[15,17]

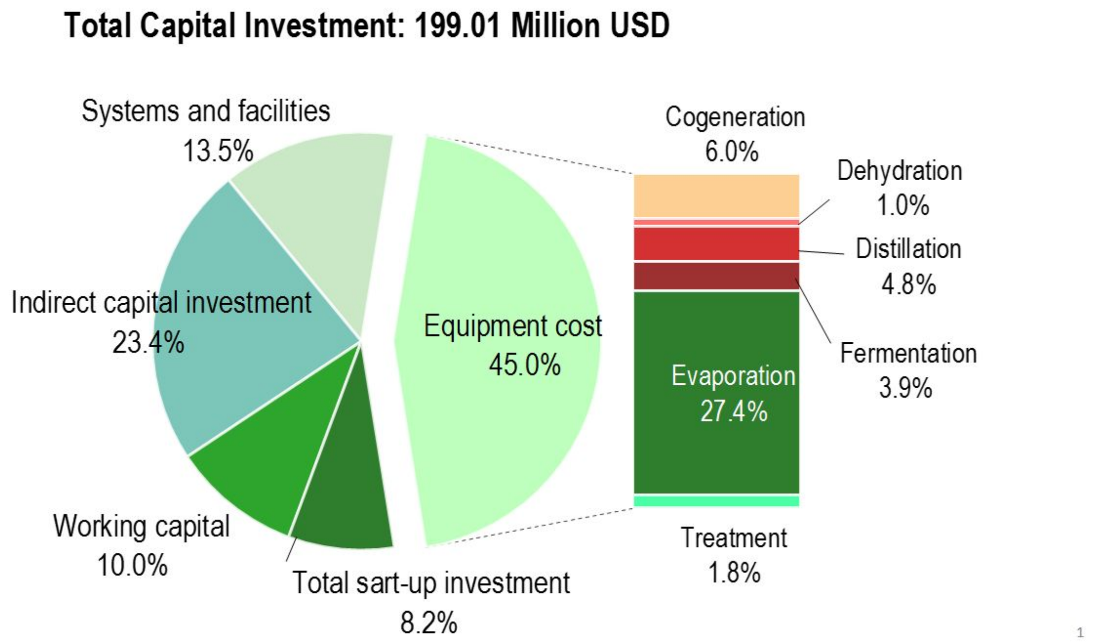

Figure S5. Contribution of categories in cost evaluation for total capital investment.

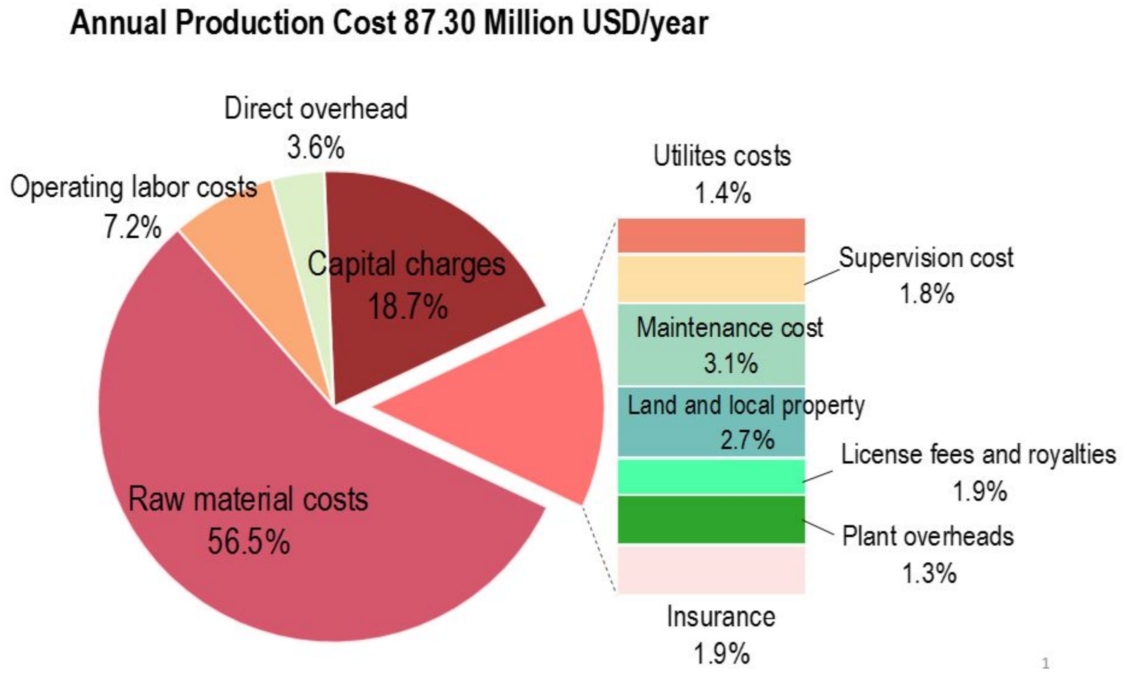

Figure S6. Contribution of categories in cost evaluation for annual production cost.

REFERENCES

(1) Popovic, M. Thermodynamic Properties of Microorganisms: Determination and Analysis of Enthalpy, Entropy, and Gibbs Free Energy of Biomass, Cells and Colonies of 32 Microorganism Species. *Heliyon* **2019**, *5* (6), e01950. https://doi.org/https://doi.org/10.1016/j.heliyon.2019.e01950.

(2) Gong, X.; Wang, S.; Qu, H. Solid-Liquid Equilibria of D-Glucose, D-Fructose and Sucrose in the Mixture of Ethanol and Water from 273.2 K to 293.2 K. *Chin J Chem Eng* **2011**, *19* (2), 217–222. https://doi.org/10.1016/S1004-9541(11)60157-2.

(3) da Luz, R. F.; Melhorim, L. O.; Cavalcante, R. M.; Young, A. F. Process Simulation Applied to the Prediction of the Impact of Corn Incorporation as Raw Material on the Brazilian Ethanol Market. *ACS Sustain Chem Eng* **2023**, *11* (28), 10352–10363. https://doi.org/10.1021/acssuschemeng.3c01339.

(4) Caudle, B.; Vieira, M. C.; Gorensek, M. B.; Chen, C. C. Modeling Phase Equilibrium of Common Sugars Glucose, Fructose, and Sucrose in Mixed Solvents. *J Chem Eng Data* **2021**, *66* (11), 4193–4205. https://doi.org/10.1021/ACS.JCED.1C00377/SUPPL_FILE/JE1C00377_SI_002.XLSX.

(5) Oliveira, L. S. B. L.; Cruz, A. J. G. Techno-Economic Analysis and Carbon Intensity of Sugarcane-Corn Flex Plants in Brazil. *Bioresour Technol Rep* **2023**, *24*, 101694. https://doi.org/10.1016/j.biteb.2023.101694.

(6) Santoyo-Castelazo, E.; Santoyo, E.; Zurita-García, L.; Camacho Luengas, D. A.; Solano-Olivares, K. Life Cycle Assessment of Bioethanol Production from Sugarcane Bagasse Using a Gasification Conversion Process: Bibliometric Analysis, Systematic Literature Review and a Case Study. *Appl Therm Eng* **2023**, *219*, 119414. https://doi.org/10.1016/J.APPLTHERMALENG.2022.119414.

(7) Joseph, A. M.; Tulasi, Y.; Shrivastava, D.; Kiran, B. Techno-Economic Feasibility and Exergy Analysis of Bioethanol Production from Waste. *Energy Conversion and Management: X* **2023**, *18*, 100358. https://doi.org/10.1016/j.ecmx.2023.100358.

(8) Le, D. M.; Nielsen, A. D.; Sørensen, H. R.; Meyer, A. S. Characterisation of Authentic Lignin Biorefinery Samples by Fourier Transform Infrared Spectroscopy and Determination of the Chemical Formula for Lignin. *Bioenergy Res* **2017**, *10* (4), 1025–1035. https://doi.org/10.1007/S12155-017-9861-4/METRICS.

(9) Bereche, R. P. Modelagem e integração energética do processo de produção de etanol a partir da biomassa de cana-de-açúcar, 2011. http://repositorio.unicamp.br/Acervo/Detalhe/803521.

(10) Morais, E. R.; Junqueira, T. L.; Sampaio, I. L. M.; Dias, M. O. S.; Rezende, M. C. A. F.; de Jesus, C. D. F.; Klein, B. C.; Gómez, E. O.; Mantelatto, P. E.; Maciel Filho, R.; Bonomi, A. Biorefinery Alternatives. In *Virtual Biorefinery: An Optimization Strategy for Renewable Carbon Valorization*; Bonomi, A., Cavalett, O., Pereira da Cunha, M., Lima, M. A. P., Eds.; Green Energy and Technology; Springer International Publishing: Cham, 2016; pp 53–132.

(11) Albarelli, J. Q.; Ensinas, A. V.; Silva, M. A. Product Diversification to Enhance Economic Viability of Second Generation Ethanol Production in Brazil: The Case of the Sugar and

Ethanol Joint Production. *Chemical Engineering Research and Design* **2014**, *92* (8), 1470–1481. https://doi.org/10.1016/J.CHERD.2013.11.016.
(12) Albarelli, J. Q.; Santos, D. T.; Ensinas, A. V.; Marechal, F.; Cocero, M. J.; Meireles, M. A. A. Product Diversification in the Sugarcane Biorefinery through Algae Growth and Supercritical CO2 Extraction: Thermal and Economic Analysis. *Renew Energy* **2018**, *129*, 776–785. https://doi.org/10.1016/J.RENENE.2017.05.022.
(13) Kamal, A.-M. Flash Separation and Distillation Columns. In *Aspen Plus*; John Wiley & Sons, Ltd, 2016; pp 99–129.
(14) Kumar, D.; Long, S. P.; Arora, A.; Singh, V. Techno-Economic Feasibility Analysis of Engineered Energycane-Based Biorefinery Co-Producing Biodiesel and Ethanol. *GCB Bioenergy* **2021**, *13* (9), 1498–1514. https://doi.org/10.1111/gcbb.12871.
(15) Sinnott, R.; Towler, G. Costing and Project Evaluation. *Chemical Engineering Design* **2020**, 275–369. https://doi.org/10.1016/B978-0-08-102599-4.00006-0.
(16) MDPyEP. *Análisis Del Complejo Productivo de La Caña de Azúcar*; La Paz, 2023. https://siip.produccion.gob.bo/repSIIP2/documento.php?n=2940.
(17) Moncada, J.; El-Halwagi, M. M.; Cardona, C. A. Techno-Economic Analysis for a Sugarcane Biorefinery: Colombian Case. *Bioresour Technol* **2013**, *135*, 533–543. https://doi.org/10.1016/J.BIORTECH.2012.08.137.
